# Nuclear Overexpression of SAMHD1 Induces M Phase Stalling in Hepatoma Cells and Suppresses HCC Progression by Interacting with the Cohesin Complex

**DOI:** 10.1002/advs.202411988

**Published:** 2024-12-16

**Authors:** Juntang Shao, Wei Wang, Shiyu Li, Guangfa Yin, Lili Han, Xinyu Wang, Meng Cai, Tao Yang, Ying Wang, Wenyan Qu, Yanhong Jiao, Peng Wang, Hanyang Xu, Xu Zhu, Songcheng Ying, Sa Xu, Qiang Sheng, Jian Fang, Tongcui Jiang, Chuansheng Wei, Yujun Shen, Yuxian Shen

**Affiliations:** ^1^ School of Basic Medical Sciences and Biopharmaceutical Research Institute Anhui Medical University 81 Meishan Road Hefei 230032 China; ^2^ Department of General Surgery The First Affiliated Hospital of Anhui Medical University 218 Jixi Road Hefei 230022 China; ^3^ Department of Hepatobiliary and Pancreatic Surgery The First Affiliated Hospital of Anhui Medical University 218 Jixi Road Hefei 230022 China

**Keywords:** cell cycle, cohesin complex, HCC, SAMHD1

## Abstract

Emerging evidence suggests that the sterile alpha‐motif (SAM) and histidine‐aspartate (HD) domain‐containing protein 1 (SAMHD1) is implicated in various cancers, including hepatocellular carcinoma (HCC). However, its precise role in tumor cells and the underlying mechanisms remain unclear. This study aimed to investigate the expression patterns, prognostic values, and functional role of SAMHD1 in HCC progression. We constructed liver tissue microarrays using tumor and paired paratumor tissue specimens from 187 patients with primary HCC. Our findings indicate that nuclear SAMHD1 protein levels are increased in tumors compared to paratumor tissues. Moreover, nuclear SAMHD1 levels decline in advanced tumor stages, with higher SAMHD1 nuclear staining correlating with favorable prognostic outcomes. Hepatocyte‐specific SAMHD1 knockout mice, generated by crossing SAMHD1^fl/fl^ mice with Alb‐cre mice, showed accelerated tumor progression in a diethylnitrosamine (DEN)‐induced HCC model. In hepatoma cell lines, nuclear overexpression of SAMHD1 inhibited cell proliferation by stalling mitosis, independent of its deoxynucleotide triphosphohydrolase (dNTPase) function. Mechanistically, SAMHD1 interacts with the cohesin complex in nucleus, enhancing sister chromatid cohesion during cell division, which delays metaphase progression. Our findings suggest that nuclear SAMHD1 plays a critical role in slowing HCC progression by regulating mitosis, highlighting its potential as a therapeutic target by manipulating cohesin dynamics.

## Graphical abstract

This study uncovers the key role for nuclear SAMHD1 in slowing hepatocellular carcinoma (HCC) progression. Elevated nuclear SAMHD1 levels in tumors correlate with favorable prognosis, and hepatocyte‐specific SAMHD1 knockout in mice accelerates tumor growth. Mechanistically, SAMHD1 interacts with the nuclear cohesin complex to stall mitosis, independent of its deoxynucleotide triphosphohydrolase dNTPase activity, positioning SAMHD1 as a potential therapeutic target in HCC.

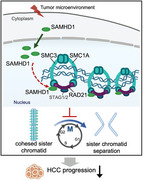

## 1 Introduction

Unbridled cell proliferation is a key hallmark of cancer.^[^ ^1^ ^]^ Cancer‐associated mutations that disrupt cell cycle regulation enable continuous cell division. However, continuous rounds of cell division result in increased reliance on other cell cycle control mechanisms in addition to cell cycle checkpoints to prevent mitotic catastrophe and maintain cell viability.^[^ ^2^ ^]^ Therefore, new findings on cell cycle control mechanisms and their role in cancer can open new perspectives on targeting cell cycle progression in cancer treatment. Hepatocellular carcinoma (HCC) is the most common type of primary liver cancer, with high mortality primarily due to the absence of suitable biomarkers for early detection, an incomplete understanding of HCC heterogeneity, and therapeutic resistance.^[^ ^3^ ^]^ Therefore, a deeper understanding of the regulatory mechanisms governing the cell cycle in tumor cells could offer new opportunities to improve outcomes for HCC patients.

The sterile alpha motif (SAM) and histidine aspartate (HD) domain containing protein 1 (SAMHD1) is a deoxynucleotide triphosphate (dNTP) hydrolase that plays a crucial role in intracellular nucleotide homeostasis.^[^ ^4^ ^]^ SAMHD1 also exhibits non‐dNTPase activities involving the binding of single‐stranded DNA or RNA.^[^ ^5^ ^]^ This functionality enables SAMHD1 to contribute to double‐strand DNA break repair, inhibit the c‐GAS/STING pathway by facilitating DNA single‐strand resection at the stalled replication fork during DNA replication,^[^ ^6^ ^]^ and restrict innate immune recognition of self‐RNA by mediating condensation of immunogenic self‐RNA via its RNase activity.^[^ ^7^ ^]^ Additionally, SAMHD1 downregulates IGF signaling and suppresses NF‐κB activation, playing a key role in the negative regulation of the innate immune response to viral infections and inflammatory stimuli.^[^ ^8^, ^9^ ^]^

Recently, SAMHD1's role in cancer has gained attention due to aberrant expression levels and mutations reported in multiple malignancies. In leukemia, SAMHD1 expression is downregulated in CD4^+^ T cells due to transcriptional promoter methylation and increased microRNA‐181 levels, which negatively regulates SAMHD1 expression.^[^ ^10^, ^11^ ^]^ In non‐small cell lung cancer patients, SAMHD1 mRNA is significantly lower in tumors compared to adjacent tissues, correlating with more aggressive tumor progression.^[^ ^12^ ^]^ In ovarian cancer, higher SAMHD1 expression is linked to poorer prognosis, whereas in cervical and colorectal cancers, SAMHD1 acts as a favorable prognostic marker.^[^ ^13^, ^14^ ^]^ In addition, mutations in SAMHD1's HD domain, which can impair or eliminate its dNTPase activity, have also been identified in various cancers.^[^ ^14^ ^]^ Despite these findings, SAMHD1's role in cancer and the underlying mechanisms remain unknown. For instance, the impact of SAMHD1 on cell proliferation presents a controversial picture: SAMHD1 knockdown inhibits the proliferation of Hela and MCF7 cells,^[^ ^15^ ^]^ while its depletion in THP‐1 cells promotes proliferation and reduces apoptosis.^[^ ^16^ ^]^ Considering SAMHD1's involvement in diverse cellular processes, such as regulating dNTP pools, maintaining genomic stability, participating in DNA repair, and immune modulation, further research is needed to better understand its role in cancer, including hepatocellular carcinoma.^[^ ^17^ ^]^

Despite SAMHD1 being a well‐known restricting factor for HIV, its function in virus infections varies by virus type. For instance, SAMHD1 plays a pro‐viral role in arbovirus infections of human skin cells.^[^ ^18^ ^]^ In the context of hepatitis B virus (HBV), SAMHD1 exhibits a dual role in virus replication. Our previous work showed that overexpression of SAMHD1 restricts HBV by inhibiting the reverse transcription of pregenomic RNA into relaxed circular DNA (rcDNA) during virus replication.^[^ ^19^ ^]^ Later, the McKeating group reported that SAMHD1, by participating in DNA repair pathways, promotes the conversion of rcDNA to the viral transcription template covalently closed circular DNA (cccDNA) during the early stages of HBV replication.^[^ ^20^ ^]^ Moreover, we observed that SAMHD1 expression is downregulated in HepG2 cells infected with HBV, potentially due to the ability of HBx to interact with DDB1, recruiting SAMHD1 to the ubiquitin E3 ligase.^[^ ^19^ ^]^ Given that HBV is a major risk factor for HCC development, these findings raise important questions about how SAMHD1 expression is altered in HCC and its role in HCC progression.

In this study, we aimed to understand the underlying mechanism and pathological significance of SAMHD1's expression changes in HCC. We constructed liver tissue microarray (TMA) chips comprising tumor tissue and paired paratumor tissue specimens from 187 cases of primary HCC. Surprisingly, our findings revealed that, regardless of HBsAg status, the nuclear level of SAMHD1 protein was elevated in tumor tissues compared to paratumor tissues, despite no difference in overall SAMHD1 expression between tumor and paratumor tissues. Notably, SAMHD1 nuclear staining intensity decreased as the tumor stage advanced, while higher SAMHD1 nuclear staining correlated with better survival outcomes, indicating its potential as a favorable prognostic marker. To explore the functional significance of nuclear SAMHD1 in HCC, we isolated the nuclear fraction of HepG2 cells and conducted immunoprecipitation coupled with liquid chromatography/mass spectrometry (IP coupled LC/MS). This analysis identified the cohesin core subunits RAD21, SMC3, and SMC1A as novel protein‐interacting partners with SAMHD1. Mechanistic studies further demonstrated that nuclear SAMHD1 overexpression restricts hepatoma cell proliferation by increasing sister chromatid cohesion, which leads to M phase stalling. Conversely, hepatocyte‐specific SAMHD1 knockout mice displayed accelerated tumor growth and migration. Collectively, our study has found a novel anti‐tumor mechanism of nuclear SAMHD1 in hepatoma cells, mediated by the prolongation of M phase through enhanced chromatid cohesion.

## 2 Results

### 2.1 Nuclear SAMHD1 Protein Levels Increased in Tumor Tissue Compared to Paratumor Tissue in HCC

**Figure 1:**
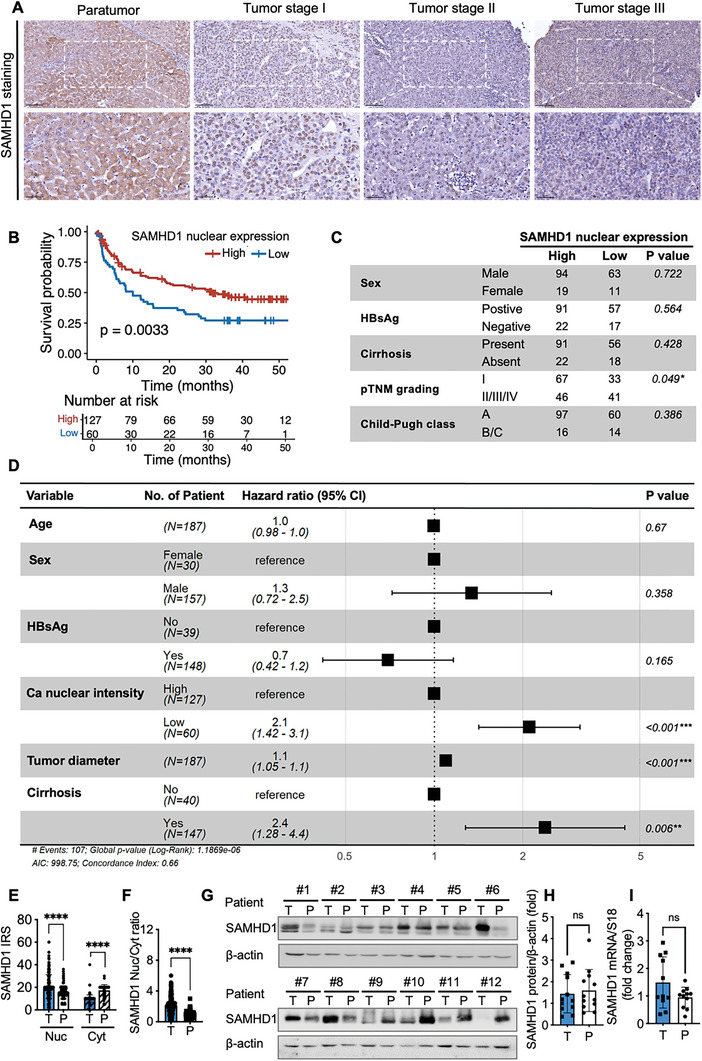
Nuclear SAMHD1 protein is increased in tumor tissues compared with paratumor tissues in HCC. (A) Representative images of IHC staining for SAMHD1 in HCC paratumor tissue and tumor tissues at TNM stages I, II, and III. Upper panel scale bar: 100 µm; lower panel scale bar: 50 µm. (B) Kaplan‐Meier survival curves exhibit significantly longer disease‐free survival (DFS) for primary HCC patients with high nuclear levels of SAMHD1, categorized according to the median Immunoreactive Score (IRS) for SAMHD1 nuclear staining (n = 187). (C) Clinicopathological correlation of nuclear SAMHD1 expression in HCC patients, pTNM, pathological tumor node metastasis. (D) Forest plot of hazard ratios (HR) for DFS according to multivariable Cox regression analysis. HRs and 95% CIs are estimated for each specified variable. (E) IRS of SAMHD1 staining results in the nucleus (Nuc) and cytoplasm (Cyt) in tumor (T) and paratumor (P) regions from 187 HCC cases. (F) The ratio of SAMHD1 IRS in the nucleus to that in the cytoplasm is significantly higher in tumor tissue compared to paratumor tissues. (G) SAMHD1 protein expression in 12 representative HCC cases. (H) SAMHD1 protein and (I) mRNA expression do not show significant differences between tumor and paratumor tissues in HCC samples. For statistical analysis, a log‐rank test and a chi‐squared test were used in B and C, respectively; Cox regression analysis was employed in D. Student's t‐tests were used for E, F, H, and I. ^*^ *p* < 0.05, ^**^ *p* < 0.01, ^***^ *p* < 0.001, ^****^ *p* < 0.0001. Data are presented as mean ± SD.

**Figure 2:**
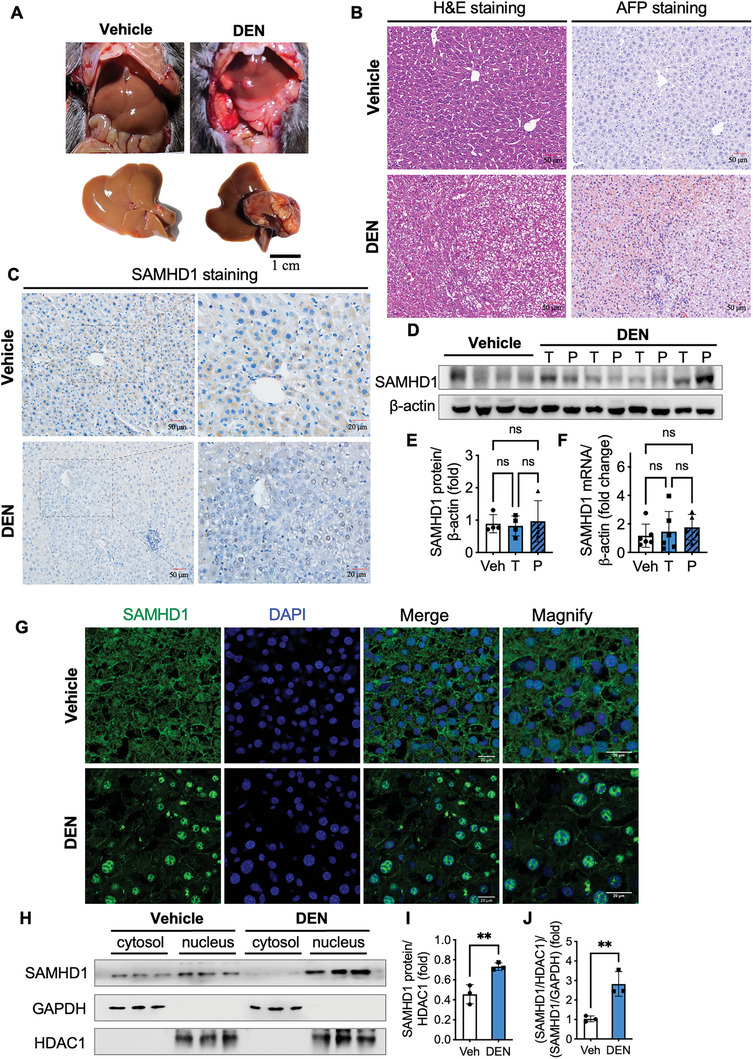
Nuclear SAMHD1 protein increased in tumor tissues of DEN‐induced HCC mouse models. (A) Representative gross liver images in wild‐type male C57BL/6J mice after 12 months of DEN or vehicle treatment. (B) Representative images of H&E staining, and IHC staining for AFP of liver slices from vehicle control‐treated or DEN‐treated mice. Scale bar = 50 µm. (C) Representative images of IHC staining for SAMHD1 of liver slices from vehicle control‐treated or DEN‐treated mice. Scale bar = 50 or 20 µm. (D) Western blot analysis of SAMHD1 in liver lysates from vehicle‐treated control mice, and in tumor (T) tissues and paratumor tissues (P) from the DEN‐induced HCC mouse model. (E, F) There are no significant differences in SAMHD1 protein and mRNA levels among the three indicated lysates. (G) Representative images of IF staining for SAMHD1 of liver slices from vehicle control‐treated or DEN‐treated mice. Scale bar = 20 µm. (H) Western blot analysis of SAMHD1 from nuclear and cytosolic fractions of tumor tissues from DEN‐induced HCC mouse model and liver tissues from control mice. (I) Nuclear SAMHD1 levels increase in tumor tissues with (J) a greater nucleus‐to‐cytoplasm ratio of SAMHD1 compared to control mice. This ratio is determined by comparing nuclear SAMHD1 levels, normalized to HDAC1, with cytosolic SAMHD1 levels, normalized to GAPDH. For statistical analysis, Student's t‐tests were used for E, F, I, and J. **P < 0.01. Data are presented as mean ± SD.

Although we have found that HBV replication reduced SAMHD1 expression in HepG2 cells,^[^ ^19^ ^]^ it is unclear how SAMHD1 expression changes in HCC, where HBV is a primary pathogenic factor. To address this, we examined SAMHD1 expression in a tissue microarray composed of 187 clinical primary HCC samples. The nuclear and cytoplasmic levels of SAMHD1 protein were quantitatively assessed based on the Immunoreactive Score (IRS). As shown in **Figure** **1**A, SAMHD1 staining was more prominent in the nuclei of tumor tissues, whereas in paratumor tissues, it was primarily localized in the cytoplasm. In addition, the levels of nuclear SAMHD1 staining decreased in tumor tissues from patients with more advanced stages of HCC (Figure 1A). Although the nuclear‐over‐cytoplasmic ratio of SAMHD1 was notably elevated in tumor tissues compared to adjacent non‐tumor tissues (Figure 1E,F), we sought to investigate whether the overall SAMHD1 protein and mRNA levels are altered in tumor tissue. We conducted western blotting and qPCR using paired tumor and paratumor tissues from surgical resections of 12 HCC patients. Both SAMHD1 protein and mRNA levels showed no significant alteration in cancerous tissues compared to paired adjacent non‐tumor tissue (Figure 1G–I). This observation remained consistent across both HBsAg‐positive and HBsAg‐negative samples, indicating that SAMHD1 expression levels might be unrelated to HBV in HCC (Figure S1B,C, Supporting Information). To rule out any potential influence of HBV infection on the observed SAMHD1 protein changes in human samples, we utilized a DEN‐induced HCC mouse model to further validate these findings. As illustrated in **Figure** **2**A,B, significant liver tumor formation is induced at 12 months post‐DEN injections. Similar to findings in clinical HCC samples, there was no significant change in SAMHD1 protein and mRNA levels in tumor tissues compared with liver lysates from vehicle‐treated control mice (Figure 2D–F). IHC and IF results of SAMHD1 staining indicated a predominant nuclear localization of SAMHD1 in HCC mouse tumor tissues, whereas in control mouse liver tissues, SAMHD1 was primarily cytoplasmic (Figure 2C,G). To quantitatively compare the nuclear and cytoplasmic distribution of SAMHD1 protein, the nuclear and cytosolic fractions of tumor tissues and normal mouse liver tissues were separated and subjected to immunoblotting for SAMHD1. The results confirmed that the nuclear SAMHD1 protein levels and the nucleus‐to‐cytosol ratio of SAMHD1 increased in tumors compared with normal liver tissues (Figure 2H–J). We also observed that nuclear SAMHD1 exhibited a more prominent increase at earlier time points post‐DEN induction (Figure S3L,M, Supporting Information), suggesting that the nuclear SAMHD1 increase may serve as an adaptive response to DEN‐induced injury.

To explore the clinical significance of nuclear SAMHD1 levels, we stratified the clinical HCC cohort into low or high groups based on the median IRS of nuclear SAMHD1. From our in‐house HCC samples, we found that higher nuclear SAMHD1 protein levels were correlated with less advanced tumor stages (Figure 1C), longer disease‐free survival (DFS) (Figure 1B), and favorable overall survival (P = 0.055, Figure S1A, Supporting Information). These data suggest that the elevated nuclear SAMHD1 levels in tumor cells predict a positive clinical outcome in HCC. Multivariable analysis confirmed the positivity for nuclear expression of SAMHD1 as an independent predictor of DFS for HCC patients (Figure 1D). Collectively, these results suggest that increased nuclear SAMHD1 levels in tumor tissue of HCC are correlated with positive clinical outcomes.

### 2.2 Nuclear Overexpression of SAMHD1 in Hepatoma Cell Lines Suppresses Cell Proliferation and Migration

**Figure 3:**
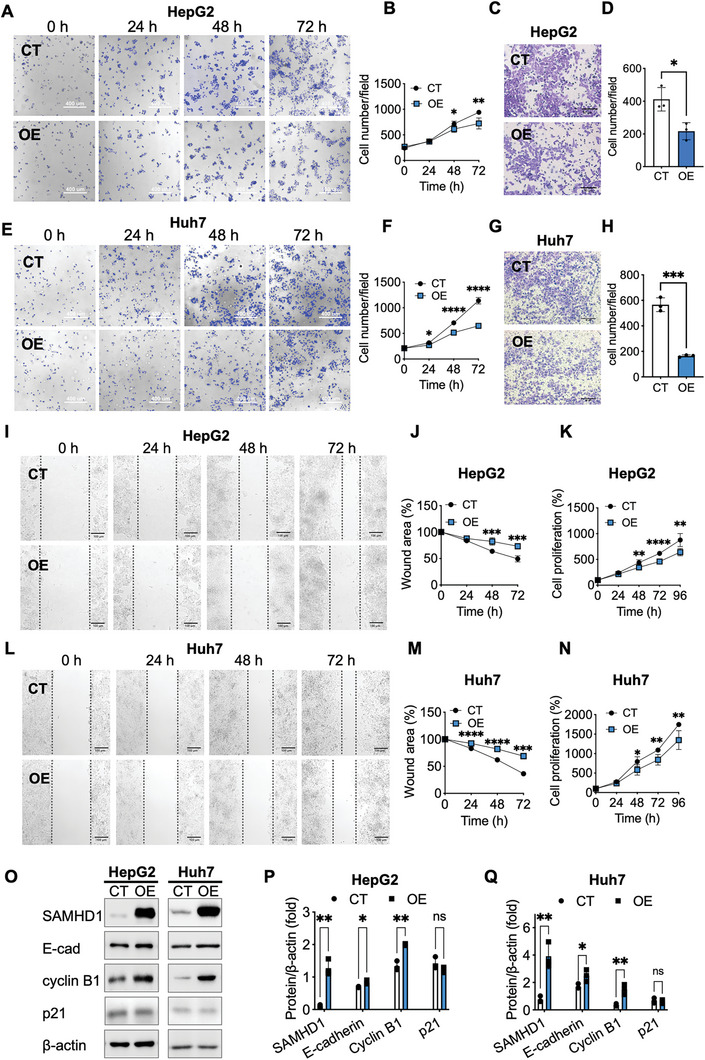
Nuclear overexpression of SAMHD1 inhibits the proliferation and migration of hepatoma cell lines. Nuclear SAMHD1 overexpression inhibits the proliferative capacity of HepG2 cells (A, B, n = 5 wells per group) and Huh7 cells (E, F, n = 4 wells per group) based on the results of Hoechest 33342 staining of nuclei over the indicated culture time. Scale bar = 400 µm. The average cell number per field was automatically calculated from 4 randomly selected regions of interest per well. SAMHD1 overexpression inhibits cell migration of HepG2 and Huh7 cells. Representative images of a transwell invasion assay and quantitative results of HepG2 (C, D) and Huh7 (G, H) cells (n = 3 wells per group), respectively. Scale bar = 50 µm. Representative images of wound healing assays and the percentage of wound closure over time are shown for HepG2 cells (I, J, n = 5 wells per group) and Huh7 cells (L, M, n = 3 wells per group), respectively. Scale bar = 100 µm. Nuclear SAMHD1 overexpression inhibits the proliferative capacity of HepG2 cells (K) and Huh7 cells (N) based on the results of the CCK‐8 assay (n = 5 wells per group). (O) WB analysis of SAMHD1, E‐cadherin, cyclin B1, and p21 in nuclear SAMHD1‐overexpressed hepatoma cell lines (OE) and control cells (CT). (P, Q) Quantification of WB results are presented in bar graphs as mean ± SD. Statistical analysis was conducted using Student's t‐tests. ^*^ *p* < 0.05, ^**^ *p* < 0.01, ^***^ *p* < 0.001, ^****^ *p* < 0.0001. Data are presented as mean ± SD. OE: SAMHD1‐overexpressed cells; CT: control cells.

To investigate the role of nuclear SAMHD1 in the proliferation and migration of hepatoma cells, we overexpressed SAMHD1 with its nuclear localization sequence in HepG2 and Huh7 cells. The nuclear localization of overexpressed SAMHD1 was confirmed by immunofluorescence staining of the HA tag, as visualized using confocal microscopy (Figure S1D,E, Supporting Information). The CCK‐8 assay and nuclear staining from live cell imaging consistently showed reduced proliferative activity in HCC cell lines with nuclear SAMHD1 overexpression (**Figure** **3**A,B,E,F,K,N). The wound healing and transwell assays demonstrated that nuclear SAMHD1 overexpression inhibited the migration of hepatoma cell lines (Figure 3C,D,G,H,I,J,L,M). Notably, overexpression of cytosolic SAMHD1, modified with the K11A mutation in its nuclear localization sequence, did not inhibit cell proliferation or migration, highlighting the specificity of nuclear SAMHD1's antitumor role (Figure S3H–J, Supporting Information). Western blotting results showed that SAMHD1 overexpression led to a significant increase in G2/mitotic‐specific cyclin B1 levels. Additionally, we found that the levels of the epithelial marker E‐cadherin were slightly increased in SAMHD1‐overexpressing HCC cell lines compared to control cells. Intriguingly, nuclear overexpression of SAMHD1 did not result in a significant change in p21, a negative regulator of cell cycle progression at the G1/S phase (Figure 3O–Q).

### 2.3 Hepatocyte‐Specific SAMHD1 Knockout Promotes Tumor Growth in DEN‐Induced HCC Model

**Figure 4:**
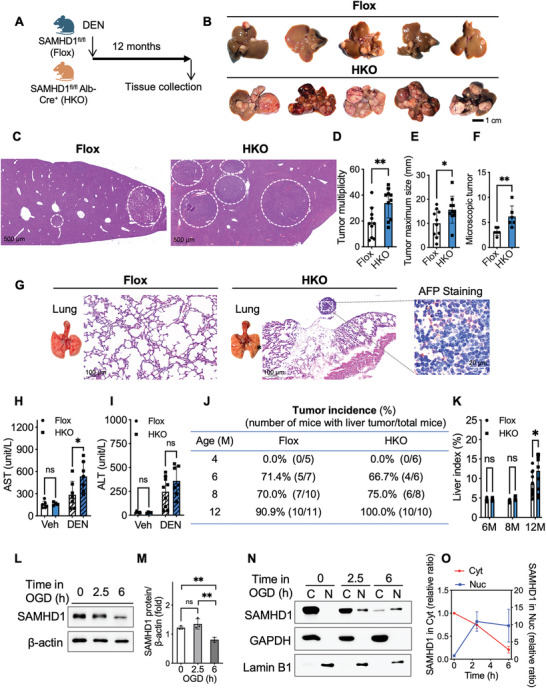
Hepatocyte‐specific SAMHD1 knockout exacerbates HCC development 12 months after DEN induction. (A) Male HKO and Flox control mice were subjected to intraperitoneal injection of DEN at 14 days post‐birth. Livers were collected and analyzed 12 months after the DEN injection. (B) Representative gross images of whole livers. (C) Representative images of H&E staining from the indicated groups after 12‐month DEN induction. Dashed circles indicate tumor nodules. Scale bar = 500 µm. The collected livers were analyzed for (D) tumor multiplicity and (E) maximum tumor size by examining visible tumors on the lobular surface, along with (F) the quantification of macroscopic tumor nodules. (G) Representative images of whole‐lung and H&E staining. Scale bar = 100 µm. The asterisk indicates a visible tumor. IHC staining for AFP confirms the HCC‐origin of tumor nodules in the lung. Scale bar = 20 µm. (H, I) Serum levels of AST and ALT were measured. (J) In a longitudinal study, tumor incidence in mice over the indicated time post‐DEN injection was quantified based on the gross examination of visible tumor nodes. (K) Liver index of the specified groups. (L, M) HepG2 cells were exposed to oxygen and glucose deprivation (OGD) for 2.5 or 6 h. SAMHD1 protein levels in whole cell lysates mildly decreased after 6 h of OGD treatment. (N) Short‐term OGD promotes the translocation of SAMHD1 from the cytoplasm to the nucleus. Cytoplasmic (C) and nuclear (N) fractions were isolated, and SAMHD1 levels were analyzed by WB, using GAPDH and Lamin B1 as cytoplasmic and nuclear markers, respectively. (O) SAMHD1 levels in the cytoplasm and nucleus were normalized to the grayscale values of the GAPDH and Lamin B1 bands, respectively. For statistical analysis, Student's t‐tests were used for D, E, F, and M; one‐way ANOVA with Tukey's post hoc tests was used for H and I; and two‐way ANOVA with Sidak's post hoc tests was used for K. ^*^ *p* < 0.05, ^**^ *p* < 0.01, data are presented as mean ± SD.

To investigate the role of hepatic SAMHD1 in the initiation and progression of HCC, we generated mice with hepatocyte‐specific conditional SAMHD1 knockout (HKO) by crossing SAMHD1^flox/flox^ mice with mice expressing hepatocyte‐specific Cre recombinase (Cre^Alb^). Abrogation of SAMHD1 expression was confirmed by the reduction in SAMHD1 protein and mRNA levels in hepatocytes, with HKO livers appearing normal histologically (Figure S2C–F, Supporting Information). Both HKO mice and littermate control mice (SAMHD1^flox/flox^) were subjected to an intraperitoneal injection of DEN on postnatal day 14 to induce HCC (**Figure** **4**A).

At 12 months post‐DEN injection, HKO mice exhibited significantly more visible tumor nodules on the liver surface compared to their control counterparts, with larger maximum tumor sizes observed in the HKO group (Figure 4B,D,E). Histological analysis further revealed that HKO mice developed more inner tumor nodules than control mice (Figure 4C,F). In addition, liver weight and liver index (calculated as the ratio of liver weight to body weight) were significantly elevated in HKO mice relative to controls (Figure 4K; Figure S2H, Supporting Information). While serum AST levels were higher in HKO mice, no significant difference in serum ALT levels was observed between the two groups (Figure 4H,I). However, at the 8‐month time point after DEN treatment, both serum AST and ALT levels were significantly elevated in HKO mice compared to Flox controls, indicating increased hepatocyte injury at this earlier stage (Figure S2I, Supporting Information). Moreover, HKO mice displayed visible tumor nodules on the pulmonary surface, with a lung metastasis rate of 2 out of 10 mice, a phenomenon not observed in the control group (Figure 4G). To investigate the role of hepatic SAMHD1 in HCC initiation, a longitudinal study was conducted at 4, 6, 8, and 12 months post‐DEN injection, tumorigenesis rates were found to be comparable between the HKO and control groups, indicating that SAMHD1 deficiency in hepatocytes did not significantly affect the initiation of DEN‐induced hepatocarcinogenesis (Figure 4J). However, the data from the 12‐month timepoint highlight a distinct role for SAMHD1 in tumor progression rather than initiation.

### 2.4 OGD Treatment Promotes Nuclear Translocation of SAMHD1 in HepG2 Cells

To investigate factors contributing to increased nuclear levels of SAMHD1 in tumor cells, we simulated the hypoxic microenvironment in proliferating HCC cells using glucose and oxygen deprivation (OGD). Total protein was extracted from HepG2 cells at various time points during OGD treatment followed by WB analysis. Compared to cells under normal conditions, SAMHD1 levels remained unchanged after 2.5 h of OGD but significantly decreased at 6 h (Figure 4L,M). To further evaluate the impact of OGD on SAMHD1's nuclear‐cytoplasmic distribution, we performed a fractionation analysis. The results showed that nuclear SAMHD1 levels increased after 2.5 h of OGD and remained comparable at 6 h, while cytoplasmic SAMHD1 gradually decreased over this period. This shift resulted in a transient increase in the nuclear‐to‐cytoplasmic ratio of SAMHD1 during short‐term OGD stress (Figure 4N,O).

### 2.5 SAMHD1 is Present in the Same Immunoprecipitated Complex with Cohesion Complex

**Figure 5:**
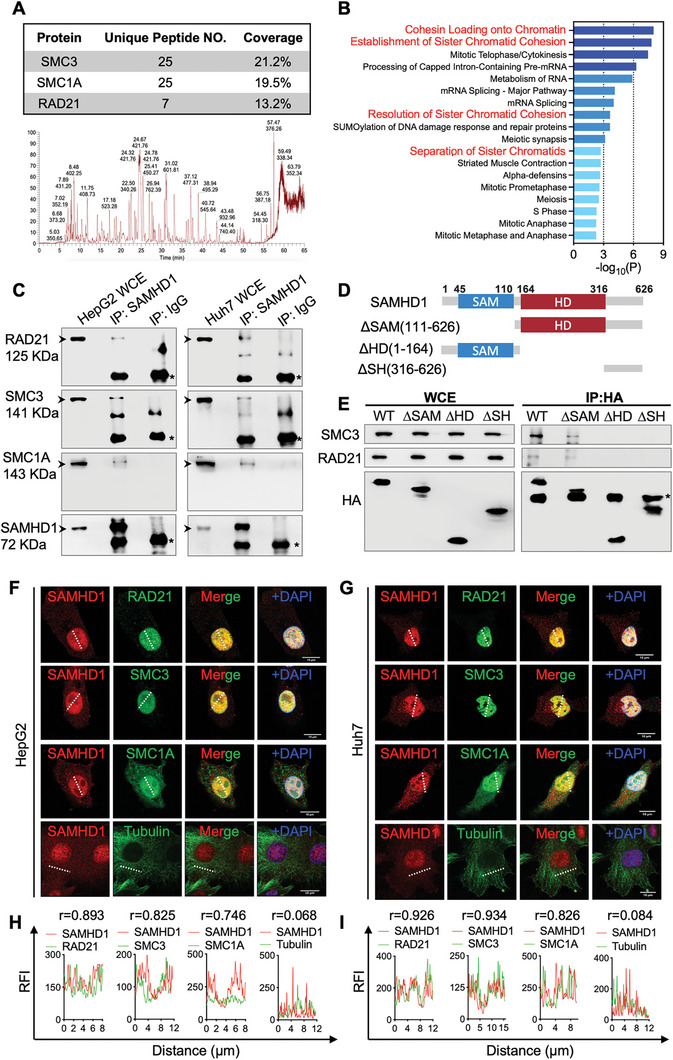
SAMHD1 interacts with the cohesin complex in the nucleus. (A) MS peak profile of immunoprecipitation‐coupled mass spectrometry (IP‐MS) using SAMHD1‐immunoprecipated HepG2 nuclear fraction. SMC3, SMC1A, and RAD21, three core components of the cohesin complex, are identified from IP‐MS. (B) Reactome pathway analysis of identified protein candidates from IP‐MS. (C) The interaction between SAMHD1 and cohesin components (RAD21, SMC3, and SMC1A) in HepG2 and Huh7 cells was validated by Co‐IP assays. Arrowheads indicate bands corresponding to the heavy chain of IgG. WCE: whole cell lysates. (D) Schematic graph of the full length and truncated SAMHD1. (E) Co‐IP analysis of the interaction between truncated SAMHD1 and cohesin components (SMC3, RAD21) in HEK293T cells. The asterisk indicates bands corresponding to the heavy chain of IgG. (F, G) Confocal microscopy images showing intracellular colocalization of SAMHD1 with cohesin complex proteins, along with negative control using tubulin to rule out bleed‐through artifacts. Representative immunofluorescence images of SAMHD1 (red) with cohesin complex components (RAD21, SMC3, SMC1A; green) in HepG2 and Huh7 cells are shown, as well as control staining of SAMHD1 (red) and tubulin (green). Scale bar = 10 µm. (H, I) Colocalization analysis was performed using Pearson correlation coefficients (r‐values) and relative fluorescence intensity (RFI) scans along the outlined traces. The RFI plots demonstrate the colocalization of SAMHD1 with cohesin proteins, while the negative control (SAMHD1 and tubulin) shows no indication of bleed‐through artifacts.

We attempted to elucidate the underlying mechanisms by which SAMHD1 affects the proliferation of hepatoma cells. As SAMHD1 displayed increased nuclear staining in HCC tumor tissues, we aimed to identify potential nuclear‐interacting partners of SAMHD1. We isolated the nuclear fraction of HepG2 cells and conducted immunoprecipitation with an anti‐SAMHD1 antibody, followed by liquid chromatography‐tandem mass spectrometry to identify SAMHD1‐interacting proteins. In total, 78 proteins were specifically identified. Reactome pathway analysis of these proteins revealed significant enrichment in multiple cohesin‐related pathways, including Cohesin Loading onto Chromatin, Separation of Sister Chromatids, and Cell Mitosis (**Figure** **5**B). Among the identified candidates, SMC3, SMC1A, and RAD21, the core components of the cohesin complex, were of particular interest (Figure 5A). Subsequent Co‐immunoprecipitation (Co‐IP) experiments confirmed the presence of SMC3, SMC1A, and RAD21 in samples co‐immunoprecipitated with SAMHD1 antibodies in both HepG2 and Huh7 cells (Figure 5C). Co‐localization studies using confocal microscopy showed pronounced nuclear co‐localization of SAMHD1 with the cohesin complex, evidenced by Pearson correlation coefficients greater than 0.7 (Figure 5F–I). These findings suggest that SAMHD1 interacts with the cohesin complex in the nucleus. To identify the SAMHD1 domains involved in this interaction, we knocked out endogenous SAMHD1 in HEK293T cells and transfected these cells with full‐length or truncated forms of SAMHD1. Co‐IP results indicated that the cohesin complex binds to the HD domain of SAMHD1 (Figure 5D,E).

### 2.6 Nuclear SAMHD1 Overexpression Leads to Increased Sister Chromatid Cohesion During Mitosis and M Phase Stalling

**Figure 6:**
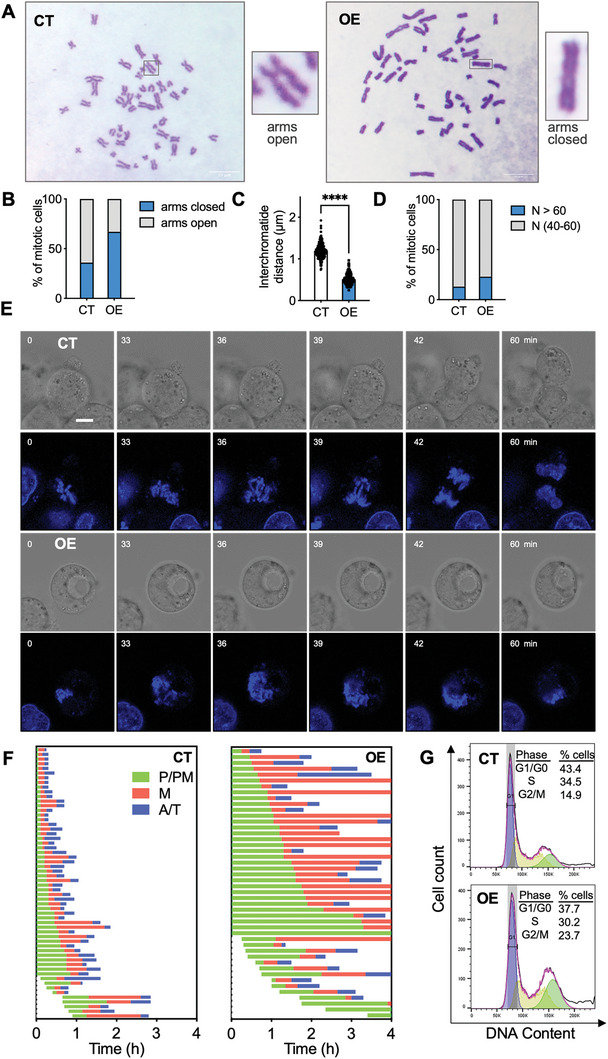
Nuclear overexpression of SAMHD1 results in over‐cohesion between sister chromatids with prolonged mitosis in HepG2 cells. (A) Representative images showing Giemsa staining of chromosome spread assays in nuclear SAMHD1‐overexpressing HepG2 (OE) and control (CT) cells. Scale bar: 10 µm. Single chromosomes (indicated by a box) are shown at higher magnification. (B) The ratio of cells with chromosome arms that were open (arms open) or closed (arms closed) was quantified and presented in bar graphs. The sample size was n = 81 for control cells and n = 73 for SAMHD1‐overexpressing HepG2 cells. (C) The bar plot shows the interchromatid distance. Over 40 cells in each group were selected randomly and the distance between sister chromatids was determined for five chromosomes in each cell. (D) The graph shows the percentage of cells with indicated chromosome number per cell. (E) M‐phase progression was observed every 3 min for 4 h using time‐lapse imaging. Representative images of CT and OE cells are shown. Scale bar = 10 µm. (F) Duration of prophase and prometaphase (P/PM, green), metaphase (M, red), and anaphase and telophase (A/T, blue) for 58 control and 46 SAMHD1‐overexpressing HepG2 cells are shown in the graph. (G) CT and OE cells were stained with PI, and the cell cycle phases were analyzed by flow cytometry, as described in the experimental section. G1/G0, S, and G2/M peaks are shown.

As the cohesin complex plays a critical role in maintaining proper sister chromatid cohesion (SCC) and separation during cell mitosis, we hypothesized that SAMHD1 might affect cell proliferation by interfering with cohesin complex‐mediated SCC. To test this hypothesis, we performed a metaphase spread assay with Giemsa staining. As shown in **Figure** **6**A, the sister chromatid arms in nuclear SAMHD1‐overexpressing HepG2 cells stayed in closer proximity, contributing to a more cohesive morphology compared to controls, which primarily displayed an “open” appearance of chromosome arms (Figure 6B). We also observed significantly shortened inter‐arm distances of sister chromatids and an increased percentage of cells with aneuploidy (chromosome numbers greater than 60 per cell) in SAMHD1‐overexpressing HepG2 cells (Figure 6C,D). Notably, SAMHD1 overexpression did not affect the protein or mRNA levels of RAD21, SMC3, or SMC1A in HepG2 cells (Figure S1F‐L, Supporting Information).

**Figure 7:**
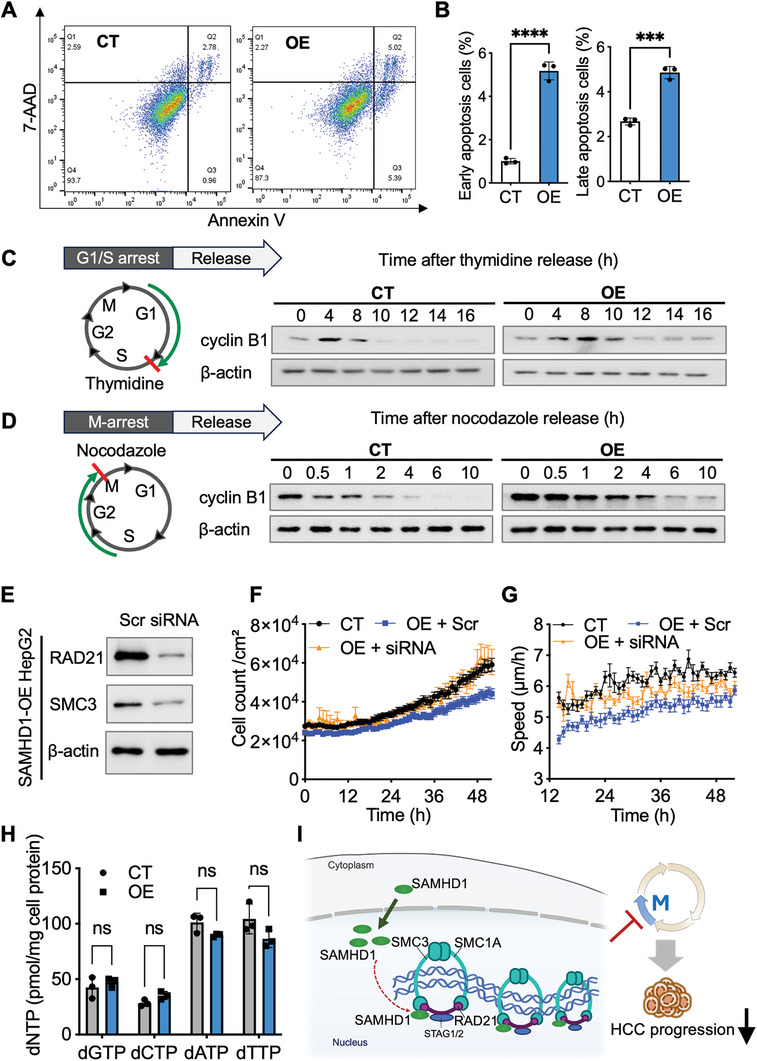
SAMHD1 stalls cell mitosis by interacting with the Cohesin complex. (A) Flow cytometry of annexin V/7‐amino‐actinomycin D (7‐AAD) staining of nuclear SAMHD1‐overexpressing HepG2 (OE) and control cells. (B) The bar plots represent the percentage of cells undergoing early and late apoptosis, respectively. Early apoptotic cells are defined as annexin V positive and 7‐AAD negative, while late apoptotic cells are both annexin V and 7‐AAD positive. (C, D) Schematic of thymidine‐ or nocodazole‐arrest and release experiment. WB analysis of cyclin B1 level released from the induced arrest for the indicated time. (E) WB analysis demonstrating the knockdown efficiency of RAD21 and SMC3 in SAMHD1‐overexpressing HepG2 cells. Scr: scrambled siRNA controls; siRNA: RAD21 siRNA and SMC3 siRNA mixture. (F, G) Cell count and cell tracking analyses of nuclear SAMHD1‐overexpressing cells after knockdown of RAD21 and SMC3 (n≥16 regions of interest per group). Data are presented as mean ± SEM. (H) Quantitation results of intracellular dNTP levels indicated that there is no significant difference between CT and OE cells. Data are presented as mean ± SD. (I) The schematic diagram illustrates that nuclear SAMHD1 restricts hepatic carcinoma progression by stalling M phase progression through increasing sister chromatid cohesion.

To investigate the impact of increased SCC induced by SAMHD1 overexpression on cell mitosis, we conducted time‐lapse imaging to continuously observe chromosome movement in the presence of Hoechst 33342 after release from RO‐3306. Representative control and SAMHD1‐overexpressing HepG2 cells are shown in Figure 6E (also see Videos S1 and S2, Supporting Information). Comparing the duration of subphase progression in the M phase between SAMHD1‐overexpressing and control cells, we found that control cells, once released from G2/M phase arrest induced by RO‐3306, completed M phase progression with an average duration of 0.85 h. In contrast, SAMHD1‐overexpressing cells exhibited increased failures in nuclear division and prolonged time in mitosis, averaging 3.8 h (Figure 6F). These data indicate that over‐cohesion caused by SAMHD1 overexpression inhibits both cohesion resolution in prophase and chromosome segregation in anaphase, leading to a prolonged M phase. Furthermore, serum re‐feeding after synchronization by serum starvation resulted in a greater accumulation of G2/M phase cells in SAMHD1‐overexpressing cells compared to controls (Figure 6G). To gain further insights into the role of SAMHD1 in mitotic progression, we performed an arrest‐and‐release experiment in control and SAMHD1‐overexpressing HepG2 cells. Cells were treated with thymidine to synchronize in the S phase and then released for WB analysis. We observed that SAMHD1‐overexpressing cells exhibited slower entry into and progression through mitosis, as evidenced by a delayed accumulation of cyclin B1 (**Figure** **7**C). In a nocodazole‐arrest‐and‐release experiment, SAMHD1‐overexpressing cells displayed delayed kinetics of cyclin B1 degradation (Figure 7D), suggesting a slowed progression from prometaphase through anaphase. Additionally, SAMHD1 overexpression increased cell apoptosis (Figure 7A,B), which might be directly attributed to prolonged mitosis or caused by aneuploidy due to the mis‐segregation of chromosomes.^[^ ^21^ ^]^ Collectively, these data indicate that nuclear SAMHD1 overexpression leads to prolonged M phase and contributes to a stalled G2/M phase in HepG2 cells.

These findings led us to ask whether over‐cohesion plays a key role in SAMHD1‐overexpression‐mediated growth inhibition. If the decrease in cell proliferation observed in SAMHD1‐overexpressing cells was indeed due to increased sister chromatid cohesion, such a phenotype would be suppressed by a reduction in the cellular level of cohesin. To test this hypothesis, we used siRNA to knock down RAD21 and SMC3 expression (Figure 7E) and found that decreased cohesin complex levels abolished the antiproliferative effects of SAMHD1 overexpression (Figure 7F; Figure S3A, Supporting Information). Additionally, the knockdown of cohesin complex components only modestly reduced the inhibitory effect of SAMHD1 on cell migration (Figure 7G; Figure S3B,C, Supporting Information), indicating that other SCC‐independent mechanisms might be involved, such as the negative regulation of the STING pathway by SAMHD1.^[^ ^12^ ^]^ Additionally, the over‐cohesion of sister chromatids and increased accumulation in the G2/M phase caused by SAMHD1 overexpression were canceled by cohesin complex knockdown (Figure S3D–G, Supporting Information). To investigate whether the impact on cell mitosis is related to SAMHD1's function as a dNTPase, we quantified the intracellular dNTP levels. The results indicated that SAMHD1 overexpression did not alter the dNTP levels in HepG2 cells (Figure 7H), which aligns with reported findings that SAMHD1 overexpression, even at very high levels, leads to only modest reductions or little impact on dNTP amounts in dividing cells.^[^ ^22^ ^]^ Furthermore, overexpression of the dNTPase‐defective SAMHD1 mutant R451E also inhibited HepG2 cell proliferation and migration, suggesting that SAMHD1's inhibitory effects on these processes are independent of its dNTPase function (Figure S3H–J, Supporting Information). Together, these results propose that SAMHD1 overexpression impedes cell proliferation by mediating the over‐cohesion of sister chromatids during mitosis.

## 3 Discussion

In this study, we observed higher nuclear SAMHD1 levels in tumor tissues from clinical HCC samples compared to paratumor tissues. Moreover, the nuclear SAMHD1 protein level serves as an independent indicator with positive prognostic values in HCC. Hepatocyte‐specific knockout of SAMHD1 exacerbates the progression of tumor growth in a DEN‐induced HCC mouse model. Additionally, nuclear SAMHD1 overexpression hinders HepG2 and Huh7 cell proliferation and migration. Collectively, these findings suggest that nuclear SAMHD1 plays an anti‐tumor role in HCC. Mechanistically, SAMHD1 interacts with cohesin complexes in the nucleus, leading to M phase stalling by increasing sister chromatid cohesion during mitosis (Figure 7I). Taken together, our findings uncovered a novel mechanism by which SAMHD1 restricts hepatoma cell proliferation, exerting control over cell mitosis progression through the modulation of cohesin dynamics.

Our study identified a distinct pattern in SAMHD1 localization in HCC, with increased nuclear expression in tumor tissues compared to paratumor tissues, despite similar overall protein levels. Notably, nuclear SAMHD1 decreases at more advanced tumor stages, and higher nuclear SAMHD1 correlates with favorable prognostic outcomes. This unique pattern appears independent of HBV, as shown by the DEN‐induced HCC mouse model, which also displayed greater nuclear SAMHD1 in tumors. Although SAMHD1 is primarily regarded as a nuclear protein with an N‐terminal nuclear localization sequence, ^11^KRPR,^14^ it is found in both the nucleus and cytoplasm of CD4^+^ T cells and macrophages.^[^ ^23^ ^]^ The nuclear import of SAMHD1 is mediated by the nuclear import receptors karyopherin α and β1.^[^ ^24^ ^]^ Elevated expression of karyopherins α1 and α2 in HCC^[^ ^25^ ^]^ may contribute to SAMHD1's increased nuclear distribution in tumors. Additionally, abnormal redox states may facilitate SAMHD1's nuclear relocalization by promoting oxidative modifications of cytoplasmic SAMHD1, enhancing its DNA‐binding capability.^[^ ^26^, ^27^ ^]^ Furthermore, our findings suggest that OGD stress promotes nuclear SAMHD1 translocation in hepatoma cells, aligning with the metabolic stress seen in solid tumors due to cancer cells’ high demand for oxygen and glucose.^[^ ^28^, ^29^ ^]^ As HCC progresses, however, nuclear SAMHD1 levels decline, shifting toward the cytoplasm. SAMHD1's nuclear export is mediated by Exportin 1 (XPO1),^[^ ^23^ ^]^ whose expression significantly increases in advanced HCC stages, as indicated by TCGA data in Figure S3K (Supporting Information). The upregulation of XPO1 may facilitate the cytoplasmic export of SAMHD1, reducing its nuclear levels in later stages. Moreover, nuclear SAMHD1 suppresses tumor cell mitosis, providing an expansion advantage to cells with lower nuclear SAMHD1 levels, which may contribute to the observed shift with tumor progression. Given these considerations, the mechanisms driving nuclear SAMHD1 increase in HCC tumors and its stage‐related nuclear decrease warrant further investigation.

Our study uncovers that nuclear SAMHD1 plays a protective role in slowing down HCC progression, supported by data showing that nuclear overexpression of SAMHD1 in hepatoma cells inhibits cell proliferation and migration. Furthermore, hepatocyte‐specific SAMHD1 knockout accelerates tumor development in DEN‐induced HCC mouse models. As we observed that nuclear SAMHD1 expression increased in tumor tissue compared to adjacent non‐cancerous tissues, it was intuitively expected that nuclear SAMHD1 may play a pro‐tumor role. However, higher nuclear SAMHD1 levels correlated with better survival outcomes in patients, suggesting a context‐dependent protective role for SAMHD1 in HCC. We propose that the elevated nuclear SAMHD1 levels in HCC may represent an adaptive response—a self‐protective mechanism of hepatocytes aimed at counteracting tumor progression, as SAMHD1 is known to participate in DNA repair pathways.^[^ ^30^ ^]^ This concept aligns with our in vitro and in vivo findings, where nuclear SAMHD1 limited HCC progression and is reminiscent of other proteins, such as ATF4, which are upregulated in tumor tissues but play tumor‐suppressive roles by maintaining cellular homeostasis.^[^ ^31^, ^32^ ^]^ Thus, nuclear SAMHD1 appears to act as a protective factor in HCC, slowing tumor progression despite its nuclear levels are elevated in tumor tissues.

We also identified that the cohesin complex as a novel group of SAMHD1‐interacting proteins in the nucleus of hepatoma cells. SAMHD1, a multifaceted protein, may manifest specific functions at different subcellular locations through interactions with context‐dependent protein partners. For instance, SAMHD1's nuclear localization is indispensable for promoting HBV cccDNA formation.^[^ ^20^ ^]^ In addition, the binding of HIV‐2 Vpx to SAMHD1 induces ubiquitination and degradation of SAMHD1, a process that also requires SAMHD1's nuclear localization sequence.^[^ ^33^ ^]^ In contrast, cytoplasmic SAMHD1 more efficiently degrades ORF2p, resulting in stronger suppression of LINE‐1 activity compared to its nuclear counterpart.^[^ ^23^ ^]^ Notably, in our study, cytosolic SAMHD1 did not inhibit HepG2 cell proliferation and migration, highlighting that the antitumor effects of SAMHD1 are specific to its nuclear localization. Furthermore, the three core components of the cohesin complex, RAD21, SMC3, and SMC1A, were identified in the same immunoprecipitants with SAMHD1 and further validated by Co‐IP assays, confirming their interaction. We also observed prominent colocalization of SAMHD1 with the cohesin complex in the nucleus, where SAMHD1 interacts with the cohesin complex via its HD domain, which is the catalytic domain of its dNTPase and is also responsible for binding to RNA or ssDNA.^[^ ^26^, ^34^ ^]^

The cohesin complex forms a ring that holds sister chromatids together until they separate at anaphase, ensuring proper chromosome segregation.^[^ ^35^ ^]^ Defects in cohesin lead to compromised chromosome segregation and chromosomal imbalance, contributing to chromosome instability, a hallmark of cancer.^[^ ^36^, ^37^, ^38^ ^]^ In cancer cells, oncogene activation and DNA replication stress weaken chromatid cohesion, increasing the risk of mitotic recombination, loss of heterozygosity, and tumor development.^[^ ^39^ ^]^ Weakened cohesion can also aid cancer cells in repairing replication forks, promoting tumor expansion.^[^ ^40^, ^41^ ^]^ Our data show that nuclear SAMHD1 overexpression causes over‐cohesion of sister chromatids, shortening inter‐arm distances and prolonging mitosis, with increased apoptosis. Cohesin complex topologically binds DNA at G1/S through an SMC3‐RAD21 gate to establish sister chromatid cohesion.^[^ ^42^ ^]^ During early prophase, cohesin dissociates from chromosome arms through Wapl‐mediated RAD21 release and ATP hydrolysis.^[^ ^43^ ^]^ In SAMHD1‐overexpressing HepG2 cells, we observed over‐cohesion, suggesting that SAMHD1 may obstruct Wapl‐mediated cleavage, reinforcing cohesin ring closure. Arrest‐release assays revealed that SAMHD1 overexpression delays mitosis, as shown by a delayed cyclin B1 peak after thymidine arrest and decelerated cyclin B1 degradation upon release from nocodazole arrest, indicating disruptions in both S to M phase transition and mitotic completion by SAMHD1 overexpression. In line with our findings, forced SMC ring closure has been shown to delay mitosis by slowing DNA replication during the S phase,^[^ ^44^ ^]^ while Wapl depletion in Hela cells leads to delayed cyclin B1 degradation, increased cohesin levels, and elongated chromosomes, resulting in a slight increase in the G2/M cell population.^[^ ^43^ ^]^ These effects mirror the changes observed with SAMHD1 overexpression, suggesting that SAMHD1 may induce similar disruptions in cell cycle progression. Collectively, our findings indicate that SAMHD1 may delay the proper opening of the cohesin ring, thereby impacting DNA replication and sister chromatid separation. Additional mechanisms, such as SAMHD1 binding to DNA and promoting cohesin loading during G1/S, cannot be ruled out.

Our study reveals a novel role of SAMHD1 in regulating cell cycle progression in hepatoma cells, extending beyond its known function as a dNTPase in maintaining cell cycle stability.^[^ ^45^ ^]^ The effect of SAMHD1's catalytic activity on cellular dNTP levels varies by cell type; while silencing SAMHD1 raises dNTP levels in non‐replicating monocyte‐derived macrophages,^[^ ^46^ ^]^ it has minimal impact in dividing HEK293T cells.^[^ ^22^ ^]^ In HepG2 cells, SAMHD1 overexpression does not reduce dNTP levels, suggesting alternative mechanisms in its inhibition of cell proliferation. This is further supported by growth inhibition observed with the overexpression of the dNTPase‐defective mutant SAMHD1 R451E. Our data indicate that SAMHD1 overexpression causes over‐cohesion of sister chromatids, reduced inter‐arm distance, and mitotic stalling with increased apoptosis. This mitotic delay, accompanied by elevated cyclin B1 and unchanged p21, suggests a p53/p21‐independent pathway, similar to effects observed in forced SMC ring closure, which bypasses ATM/p53/p21 regulation.^[^ ^44^ ^]^ Additionally, reducing cohesin levels canceled SAMHD1's inhibitory effects on HepG2 cell growth and alleviated the over‐cohesion of sister chromatids as well as the increased G2/M phase accumulation caused by SAMHD1 overexpression, suggesting that SAMHD1 modulates cell proliferation through its interactions with the cohesin complex. Normally, most cohesin dissociates from chromosome arms in mitotic prophase to enable sister‐chromatid separation, while persistent cohesion disrupts mitosis. Consistent with our findings, changes in cohesin factors like Separase, STAG2, and SGO1 are linked to cell cycle disruptions.^[^ ^41^ ^]^ These results highlight SAMHD1 as a modulator of cohesin dynamics, orchestrating cell cycle regulation in hepatoma cells.

Our study found that hepatocyte‐specific SAMHD1 knockout did not affect tumorigenesis rates in the DEN‐induced HCC model, suggesting SAMHD1 plays a limited role in tumor initiation. Previous studies also reported no increase in spontaneous tumor formation in SAMHD1‐deficient mice, possibly due to low DNA damage levels controlled by p53‐mediated responses, indicating that SAMHD1 knockout is not linked to a strong mutator phenotype.^[^ ^47^ ^]^ We propose that the growth inhibition seen with SAMHD1 overexpression results from gradual cell cycle slowing, rather than an acute effect typical of strong cell cycle inhibitors. Our in vitro data suggest that nuclear SAMHD1 slows the cell cycle by stalling mitosis, leading to reduced proliferation, and studies in HKO mice showed only modestly accelerated tumor progression post‐DEN induction. Together, these findings support SAMHD1's role as a modulator rather than an acute cell cycle checkpoint in tumor cells. The association of higher nuclear SAMHD1 with less advanced tumor stages and improved disease‐free survival highlights its potential as a long‐term therapeutic factor in HCC.

This study demonstrates that nuclear overexpression of SAMHD1 increases SCC, leading to M phase stalling. However, in addition to cell mitosis, the cohesin complex is also crucial for DNA double‐strand break repair and the topological regulation of DNA transcription, such as in interferon gamma signaling responses.^[^ ^48^, ^49^ ^]^ The fact that many cancer cells with cohesin mutations remain euploid suggests that cohesin dysfunction may drive cancer through mechanisms beyond chromosomal segregation errors, such as DNA damage in cancer‐related genes or transcriptional dysregulation.^[^ ^50^ ^]^ Given that SAMHD1 deficiency also impairs DNA repair^[^ ^30^ ^]^ and activates interferon signaling pathways,^[^ ^7^ ^]^ SAMHD1's interaction with cohesin complex may have broader implications in cancer progression beyond cell cycle control, warranting further investigation.

Cohesin cancer biology has attracted growing research interests in cancer pathogenesis and in opportunities for exploiting these findings for the clinical benefit of cancer patients. In conclusion, we reported new findings of increased SAMHD1 protein levels in the nucleus in HCC and its positive prognostic value. Moreover, we discovered a new mechanism of SAMHD1 in tuning the cell cycle of hepatoma cells, which might be a new therapeutic strategy to restrain tumor progression by mediating cohesin complex dynamics during cell mitosis.

## 4 Experimental Section

### Cell Culture and Plasmids

HEK293T cells (GNHu44), HepG2 cells (TCHu72) and Huh7 cells (TCHu182) were obtained from Cell Bank of Chinese Academy of Sciences and cultured in DMEM (C11995500BT, Gibco) supplemented with 10% fetal bovine serum (C04001, VivaCell), penicillin−streptomycin (C0222, Beyotime), and 2 mM L‐glutamine (C0212, Beyotime). Cells were grown on tissue culture dishes or in multiwell plates (Costar) at 37 °C and 5% CO₂. Primary mouse hepatocytes were isolated using a two‐step collagenase approach.^[^ ^51^ ^]^ Nuclear SAMHD1‐overexpressing HepG2 and Huh7 cells were constructed using the lentivirus transduction method using expression vector pLVX‐HA‐SAMHD1‐IRES‐Puro plasmid. HepG2 and Huh7 cells transduced with lentivirus encapsulating the backbone of expression vector pLVX IRES‐Puro plasmid were used as control cells. SAMHD1‐knockout (KO) HEK293T cells were constructed by transfecting cells with SAMHD1 sgRNA PX260 plasmid using Lipofectamine 2000 reagent (11668027, Invitrogen) with subsequent puromycin selection (2 µg mL^−1^). Then SAMHD1‐KO HEK293T cells were transfected with pcDNA3.1 vector coding for full‐length or different truncates of SAMHD1(ΔHD: SAMHD1 1–164, ΔSAM: SAMHD1 111–626, ΔSH: SAMHD1 316–626) using lipo2000‐mediated transfection. In knockdown assays, cells were transfected with siRNA sequences targeting SAMHD1 using the Lipofectamine RNAiMAX transfection reagent (13778100, Invitrogen) following the manufacturer's protocol. The siRNA and sgRNA sequences are provided in Table S1 (Supporting Information). Detailed information about antibodies, including their sources and catalog numbers, is provided in Table S2 (Supporting Information).

### Animal Studies

C57BL/6J mice (6–8 weeks, 20–25 g) were obtained from Jiangsu Jicui Yaokang Biotechnology Co., Ltd. SAMHD1^flox/flox^ mice on the C57BL/6J background bearing loxP sites flanking exon 3 of the SAMHD1 gene were cross‐bred with Alb‐cre mice to generate hepatocyte‐specific SAMHD1 knockout mice. The efficiency of SAMHD1 knockout in HKO mouse livers and hepatocytes was validated by evaluating Cre‐mediated recombination and SAMHD1 protein levels (Figure S2B–F, Supporting Information). Mice were maintained under specific pathogen‐free conditions with a standard 12‐h light cycle. They had ad libitum access to distilled water and rodent chow (Shoobree, Nanjing, China). At postnatal day 14, male mice were subjected to the induction of an in‐situ liver cancer model through intraperitoneal injection of 30 mg kg^−1^ diethylnitrosamine (HY‐N7434, MCE). Euthanasia was performed at 4, 6, 8, and 12 months after DEN administration. Liver and lung tissues were collected for subsequent analyses. Whole blood was collected through cardiac puncture, and serum was obtained following centrifugation at 2000 g for 10 min.

### Tissue Microarray and Survival Analysis

Clinical samples were obtained with informed consent from 187 HCC patients who underwent surgery between 2016 and 2018 at the First Affiliated Hospital of Anhui Medical University (AHMU). Clinicopathological characteristics are summarized in **Table** **1**. Tissue microarrays were constructed from paraffin‐embedded cancerous and adjacent non‐tumor tissues. SAMHD1 immunohistochemistry was performed on 4 µm sections, followed by hematoxylin staining of nuclei. Digital images were captured using a 3DHISTECH Pannoramic MIDI slide scanner. Two pathologists, blinded to patient information, independently assessed SAMHD1 positivity in both the cytoplasm and nucleus across five random fields per sample. Immunoreactivity scores (IRS = staining intensity × positive rate) were used to evaluate SAMHD1 expression, with intensity scored as 0 (no color), 1 (pale yellow), 2 (tan‐yellow), and 3 (brown), and the positive rate as 0 (negative) to 4 (≤10%, 11%‐50%, 51%‐75%, 76%‐100%). For survival analysis, the overall survival and disease‐free survival data of the 187 patients were used to assess the prognostic value of nuclear SAMHD1 expression. Patients were stratified into high and low‐expression groups based on the median nuclear SAMHD1 staining intensity. Kaplan‐Meier survival curves with log‐rank tests and Cox proportional hazard models were used, utilizing R (version 4.2.2), RStudio, and packages including survival, survminer, and ggplot2. The correlation between nuclear SAMHD1 expression and clinicopathological features such as sex, hepatitis B surface antigen status, cirrhosis, TNM stage, and Child‐Pugh class was analyzed using the Chi‐squared test.

**Table 1:** Clinicopathological characteristics of HCC patients.

Characteristics	Median (25th – 75th percentile)
Age	56 (47.5 – 64.5)
Characteristics	Number of Cases (%)
Sex	
Male	157 (84)
Female	30 (16)
Recurrence and metastasis	
Yes	107 (57.2)
No	80 (42.8)
pTNM grading	
I	100 (53.5)
II/III/IV	87 (46.5)
Child‐Pugh score	
A	157 (84)
B	29 (15.5)
C	1 (0.5)
HBsAg	
Negative	39 (20.9)
Positive	148 (79.1)
HCV Ab	
Negative	183 (97.9)
Positive	4 (2.1)
Cirrhosis	
No	40 (21.4)
Yes	147 (78.6)
High blood pressure	
No	154 (82.4)
Yes	33 (17.6)
Diabetes	
No	167 (89.3)
Yes	20 (10.7)
Smoking	
No	134 (71.7)
Yes	53 (28.3)
Alcohol drinking	
No	146 (78.1)
Yes	41 (21.9)

Cirrhosis is defined as a condition of severe liver scarring and impaired function, diagnosed using clinical, endoscopic, histological, and imaging criteria, as outlined in the Chinese guidelines on liver cirrhosis management (2019). High blood pressure is defined as a consistent systolic pressure of ≥140 mmHg and/or a diastolic pressure of ≥90 mmHg. Diabetes is defined by fasting blood glucose levels ≥7.0 mmol L^−1^, 2‐h postprandial glucose levels ≥11.1 mmol L^−1^, or HbA1c ≥6.5%. Smoking is defined as a history of smoking at least one cigarette daily for more than 1 year. Alcohol drinking is defined as habitual consumption over 5 years, with an ethanol intake of ≥40 g day^−1^ for men or ≥20 g day^−1^ for women.

### Cell Proliferation, Migration, and Mobility Assay

Cell proliferation was evaluated using the CCK‐8 assay and Hoechst 33342 nuclear staining. Cells were seeded in 96‐well plates at 5000 cells per well and allowed to adhere overnight. At specified time points, CCK‐8 reagent (AC11L054, Life‐iLab) was added and incubated for 2 h at 37 °C, following the manufacturer's instructions. Absorbance was measured at 450 nm using a microplate reader (Supermax 3100, Flash) to determine relative cell proliferation. For nuclear staining, cells were similarly seeded at 5000 cells per well, washed with PBS, and stained with 1 µg mL^−1^ Hoechst 33342 (BL803A, Biosharp) for 15 min at 37 °C. After washing to remove excess dye, images of stained nuclei were captured using the ImageXpress Micro Confocal System (Molecular Devices). A minimum of 4 fields per well were randomly selected, and nuclei were counted using the Cell Count module in the accompanying analysis software.

Cell migration was evaluated using a wound healing assay and a Transwell assay. For the wound healing assay, cells were seeded in 6‐well plates and grown to confluence. A scratch was made with a sterile pipette tip, followed by washing to remove detached cells. Wound closure was monitored over time using phase‐contrast microscopy, and images were analyzed with ImageJ to calculate the percentage of wound closure relative to the initial area. For the Transwell assay, cells were resuspended in serum‐free DMEM at a density of 7.5 × 10⁴ cells mL^−1^. A total of 200 µL of this suspension was added to the upper chamber, with 500 µL of DMEM containing 20% FBS in the lower chamber. After 24 h of incubation, migrated cells were fixed with 4% paraformaldehyde and stained with 0.1% crystal violet.

To assess the impact of cohesion complex knockdown on the proliferation and migration of SAMHD1‐overexpressing HepG2 cells, cells were suspended in a medium containing a mixture of RNAiMax transfection reagent with scrambled siRNA or RAD21 and SMC3 siRNA mixture and were seeded in 6‐well plates at 20% confluence for 24 h. Then the cell plates were placed into the HoloMonitor M4 digital holographic microscopy (Phase Holographic Imaging PHI Inc) within a standard CO₂ incubator. Six regions of interest (ROI) in each well were randomly selected by the instrument software, and images were captured every 2 h for a continuous 52‐h period. The HoloMonitor software's Kinetic Cell Proliferation Assay and Kinetic Cell Motility Assay modules were used to quantify cell growth and calculate average migration speed based on the captured images.

### Immunoprecipitation and Proteomics

Nuclear fractions of HepG2 cells were prepared according to the manufacturer's manual (Nuclear Complex Co‐IP Kit, Active Motif company). Immunoprecipitation was conducted using HepG2 nuclear extracts with an anti‐SAMHD1 antibody (12586‐1‐AP, Proteintech) or isotype control antibody (B900610, Proteintech). Proteins were subjected to SDS−PAGE separation following silver staining (P0017S, Beyotime), and the excised gel was sent to Shanghai Applied Protein Technology Company for downstream analysis. In brief, LC−MS (Q Exactive Quadrupole‐Orbitrap Mass Spectrometer and Easy‐nLC 1000 chromatograph, Thermo Fisher) was performed on the peptide mixture obtained from trypsin digestion of protein samples. The peptide sequence was identified by bioinformatics analysis using MASCOT software. Unique peptides of Cohesin complex RAD21, SMC1A, and SMC3 were identified by mass spectrometry and are listed in Figure 5A.

### Metaphase Spread Assay

Cells were treated with 100 ng ml^−1^ Colcemid (HY‐N0282, MCE) for 1.5 h to induce M phase arrest, then collected and resuspended in 0.075 mM KCl solution at 37 °C for 15 min. Cells were then fixed with a methanol‐acetic acid solution (volume ratio 3:1) and dropped onto glass slides. After staining in 5% Giemsa solution (C0133, Beyotime), images were captured using an Axioscope 5 microscope. Chromosome analysis was conducted using Fiji ImageJ software, wherein the number of chromosomes in cells undergoing mitosis was counted using the plugin Levan. Additionally, the software was utilized to measure the interarm distance between sister chromatids using the macro InteredgeDistance.

### Flow Cytometry and Live Cell Imaging

Cells were seeded into 6‐well plates and subject to serum‐free starvation for 24 h. Following starvation, cells were restimulated with DMEM containing 10% FBS for another 24 h. Subsequently, cells were collected, fixed in pre‐chilled 75% ethanol, washed with pre‐chilled PBS, and stained with 200 µL propidium iodide (PI) solution (HY‐D0815, MCE) containing RNase A (ST579, Beyotime) for 15 min. The stained cells were then analyzed using a flow cytometer (FACS Celesta, BD). Cell cycle distribution was analyzed using FlowJo software.

Cells were cultured in DMEM with 10% FBS on glass‐bottom dishes and treated with 4 µm RO‐3306 (R275210, Aladdin) for 20 h to induce G2 phase arrest. Following treatment, cells were replenished with fresh DMEM containing 0.1 µm Hoechst 33342 for live imaging of chromosome changes, mitosis, and M phase duration. Confocal microscopy (Zeiss 980) and automated live cell imaging (Zeiss Cell Discoverer 7.0) were used at 37 °C with 5% CO₂, capturing images every 3 min for up to 4 h. The duration of prophase/prometaphase was measured from mitotic entry (chromosome condensation and cell rounding) to metaphase (chromosome alignment), followed by metaphase (from alignment to chromosome separation) and anaphase/telophase (from chromosome separation to cleavage furrow completion).

For apoptosis analysis, cells treated with RO‐3306 were allowed to grow for an additional 12 h in fresh DMEM before staining with 7‐AAD and Annexin V (Annexin V‐APC/7‐AAD Apoptosis Kit, Multi Sciences). Apoptosis was analyzed using flow cytometry (FACS Celesta, BD).

### Intracellular dNTP Determination

Cells were plated in 10‐cm tissue culture dishes and allowed to adhere and enter log‐phase growth. The adherent cells were detached by trypsin and resuspended gently in 10 ml of ice‐cold PBS. The samples were centrifuged for 5 min at 3000 g at 4 °C, the supernatant discarded, and cell pellets were then resuspended in 500 µl of ice‐cold 60% methanol, vortexed vigorously to resuspend, placed at 95 °C for 3 min and sonicated for 30 s. The extracts were centrifuged (16000 g for 5 min at 4 °C) to remove cell debris, precipitated protein, and DNA. The resultant cell extracts were evaporated under a centrifugal vacuum at 70 °C and the resultant pellet was resuspended in 25 µL nuclease‐free water ready to assay. Cellular dNTPs were quantified using a fluorescence‐based assay on a real‐time PCR thermocycler (QuantStudio 5, Thermo Fisher) as described.^[^ ^52^ ^]^

### Study Approval

Ethics approval (PJ 2021‐12‐29) was obtained from the First Affiliated Hospital of Anhui Medical University (Hefei, China) to use the clinical samples for research purposes. All patient tissue samples were collected with informed consent. The animal studies were approved by the Animal Care and Use Committee of Anhui Medical University (LLSC20240908).

### Statistical Analysis

Experimental data were analyzed with the PRISM statistical package. If not stated otherwise, all data were normally distributed and expressed as mean ± SD. For comparisons, the chi‐squared test, one‐way analysis of variance with ad hoc Tukey test, and two‐tailed Student *t*‐test were performed as appropriate in Excel or GraphPad Prism (v10).

## Conflict of Interest

The authors declare no conflict of interest.

## Supporting information



Supporting Information

Supplemental Video 1

Supplemental Video 2

## Data Availability

The data that support the findings of this study are available from the corresponding author upon reasonable request.
