# Supplementary material for: Nuclear Overexpression of SAMHD1 Induces M Phase Stalling in Hepatoma Cells and Suppresses HCC Progression by Interacting with the Cohesin Complex
Source: Adv Sci (Weinh). 2024 Dec 16;12(6):2411988. doi: 10.1002/advs.202411988 (PMC11809348; doi:10.1002/advs.202411988)
Supplement: Supplementary file 1 — Supporting Information [file ADVS-12-2411988-s003.docx]

**Supplementary figures**


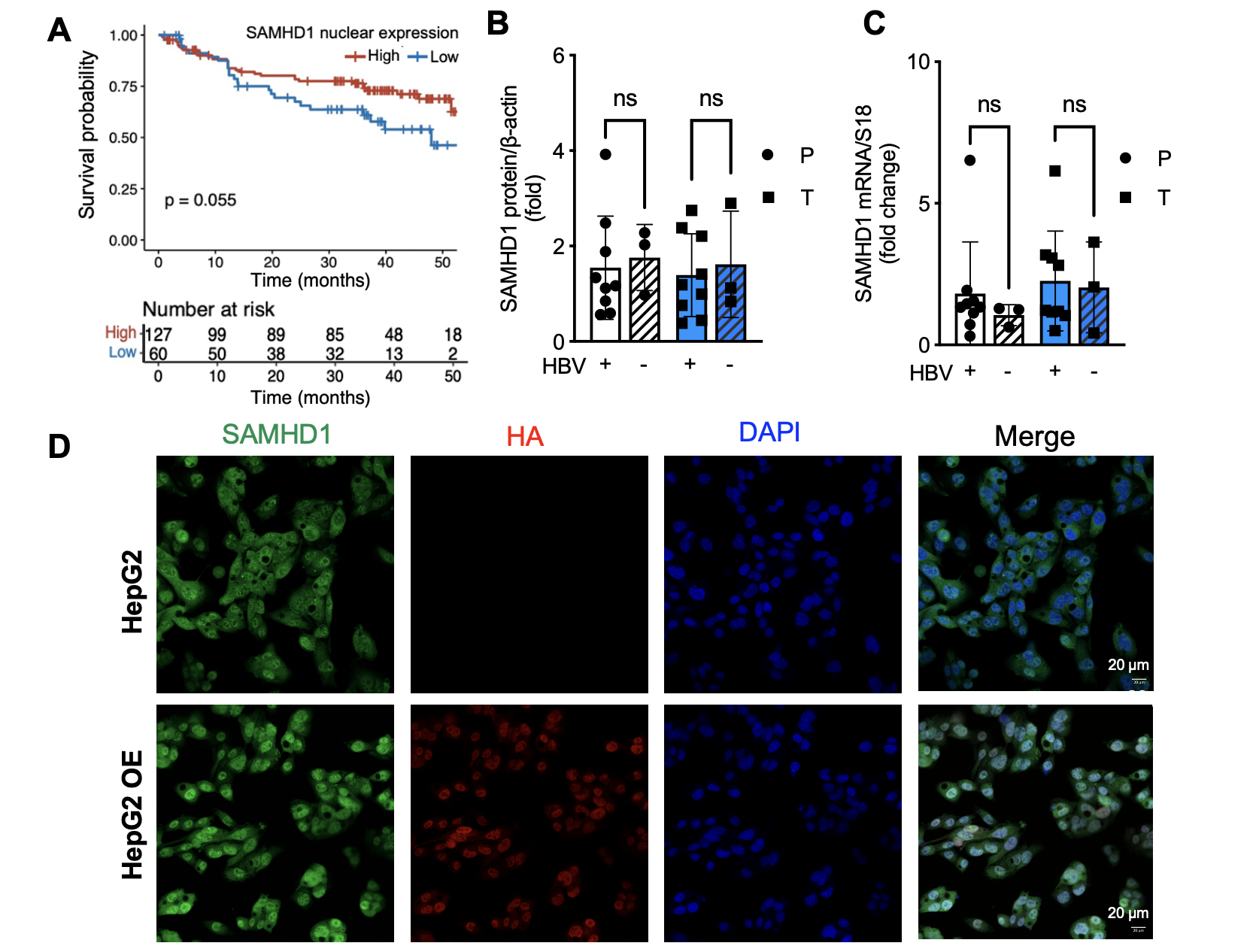

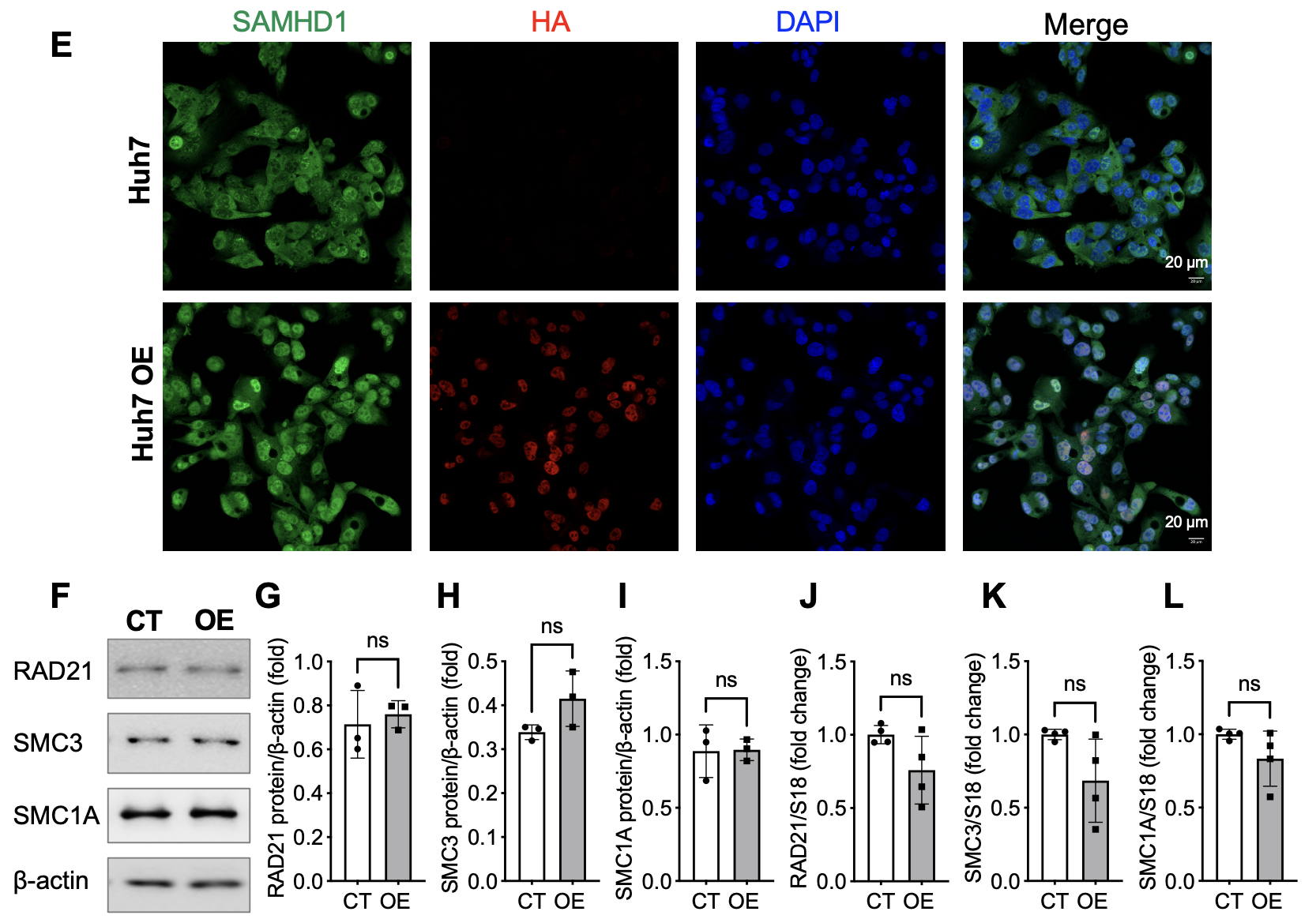


**Figure S1.** (A) Kaplan-Meier survival curves of overall survival (OS) in relation to nuclear SAMHD1 staining. (B) Quantitative results of SAMHD1 protein detected in tumor (T) and paratumor (P) tissues from HCC patients with or without HBV. WB results are presented in Figure 1G. (C) SAMHD1 mRNA detected by qPCR in tumor and paratumor tissues from HCC patients with or without HBV. (D, E) Immunofluorescence imaging confirms the nuclear localization of overexpressed SAMHD1 in HepG2 and Huh7 cells, as indicated by HA tag detection. Scale bar =20 µm. (F) SAMHD1 overexpression does not result in significant changes in RAD21, SMC3, or SMC1A protein levels in HepG2 cells. (G-I) Quantitative results of WB. (J-L) SAMHD1 overexpression does not result in significant changes in RAD21, SMC3, or SMC1A mRNA levels in HepG2 cells.


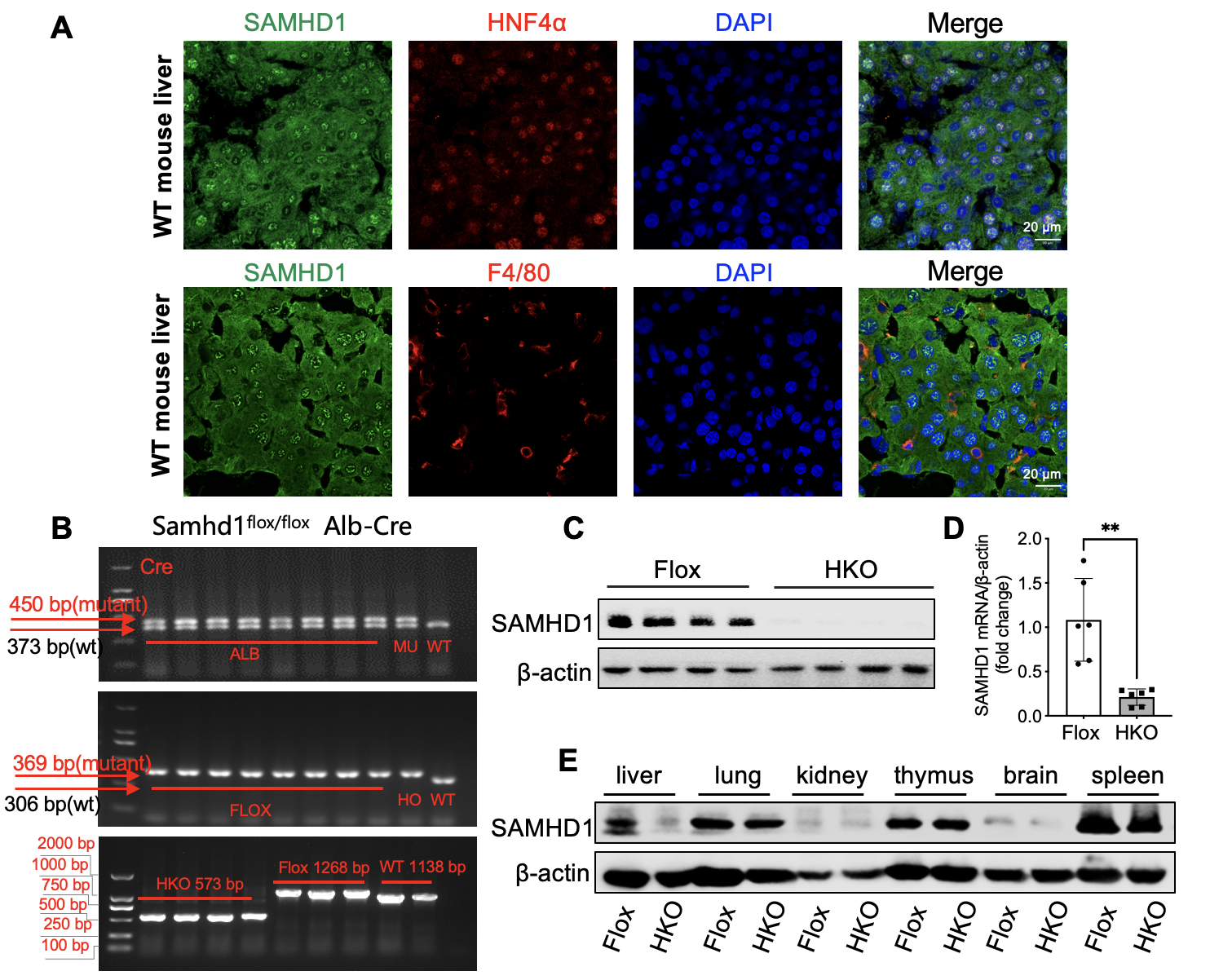

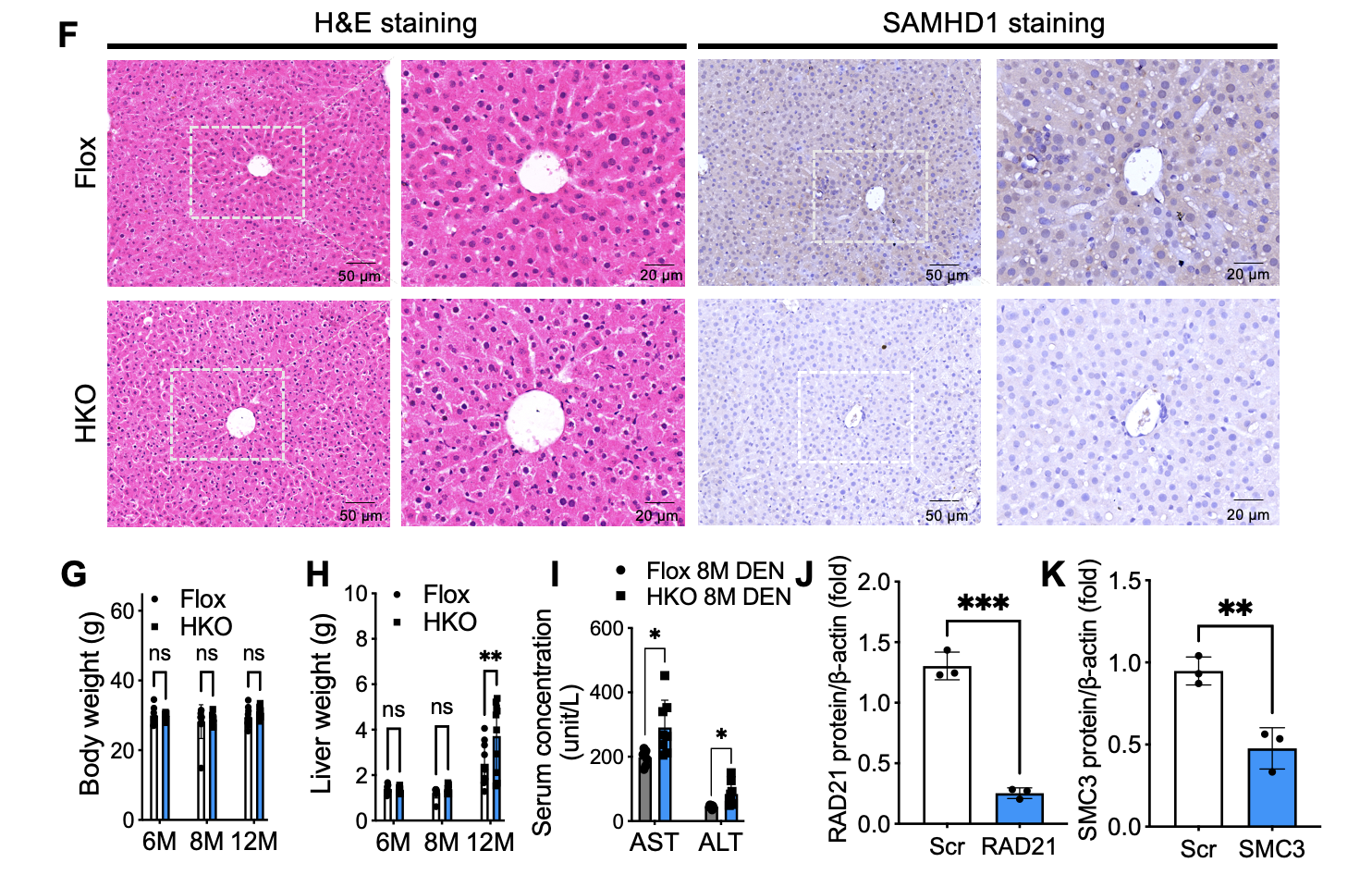


**Figure S2.** (A) SAMHD1 is expressed in both hepatocytes and macrophages in mouse liver. Representative images of double immunofluorescence staining using SAMHD1 along with HNF4α (a hepatocyte marker) or F4/80 (a macrophage marker). (B) SAMHD1^flox/flox^ mice on the C57BL/6J background bearing loxP sites flanking exon 3 of the SAMHD1 gene were cross-bred with Alb-cre mice to generate hepatocyte-specific SAMHD1 knockout (HKO) mice. Primers used with genomic DNA from tail biopsy for mouse genotyping are listed as follows: the P1 (5’-TGGCAAACATACGCAAGGG-3’) and P2 (5’-CGGCAAACGGACAGAAGCA-3’) primers generate a 450-bp band in Alb-cre mice. The P1 and Alb promoter (5’-CTAGAAATCAGCACTAAAGGAA-3’) primers generate a 373-bp band in mice with a WT Alb promoter. For detecting loxP insertion, the P3 (5’-CGGGGCTTGGGGTTAGTTTCTATTTCA-3’) and P4 (5’-ACCCCAGAACCTCCAGCCACATACAGA-3’) primers generate a 369-bp band in loxP inserted mice and 306-bp corresponding to WT. For detecting Cre activity in hepatocytes, the primers P5 (5’-TCAGAGTGTCAGGCAGATTGTGGTAAAGA-3’) and P6 (5’-CCCAGAACCTCCAGCCACATACAGAAATA-3’) were used with genomic DNA extracted from liver tissues. The targeted allele produces a 1138-bp band and 1268-bp in WT and SAMHD1^flox/flox^ mice, respectively, while after recombination, the primers produce a 573-bp PCR product. (C) SAMHD1 protein level in primary hepatocytes detected by WB. (D) SAMHD1 mRNA in primary hepatocytes detected by qPCR. (E) SAMHD1 protein in indicated tissues from Flox and HKO mice. (F) Representative images of H&E staining and SAMHD1 IHC results of liver sections from HKO and Flox control mice. (G, H) Body weight and liver weight of Flox and HKO mice after DEN induction at the indicated time. (I) Serum levels of AST and ALT were measured from Flox and HKO mice 8 months after DEN induction. (J, K) Quantitative results of siRNA knockdown efficiency. Representative WB results are presented in Figure 7E.


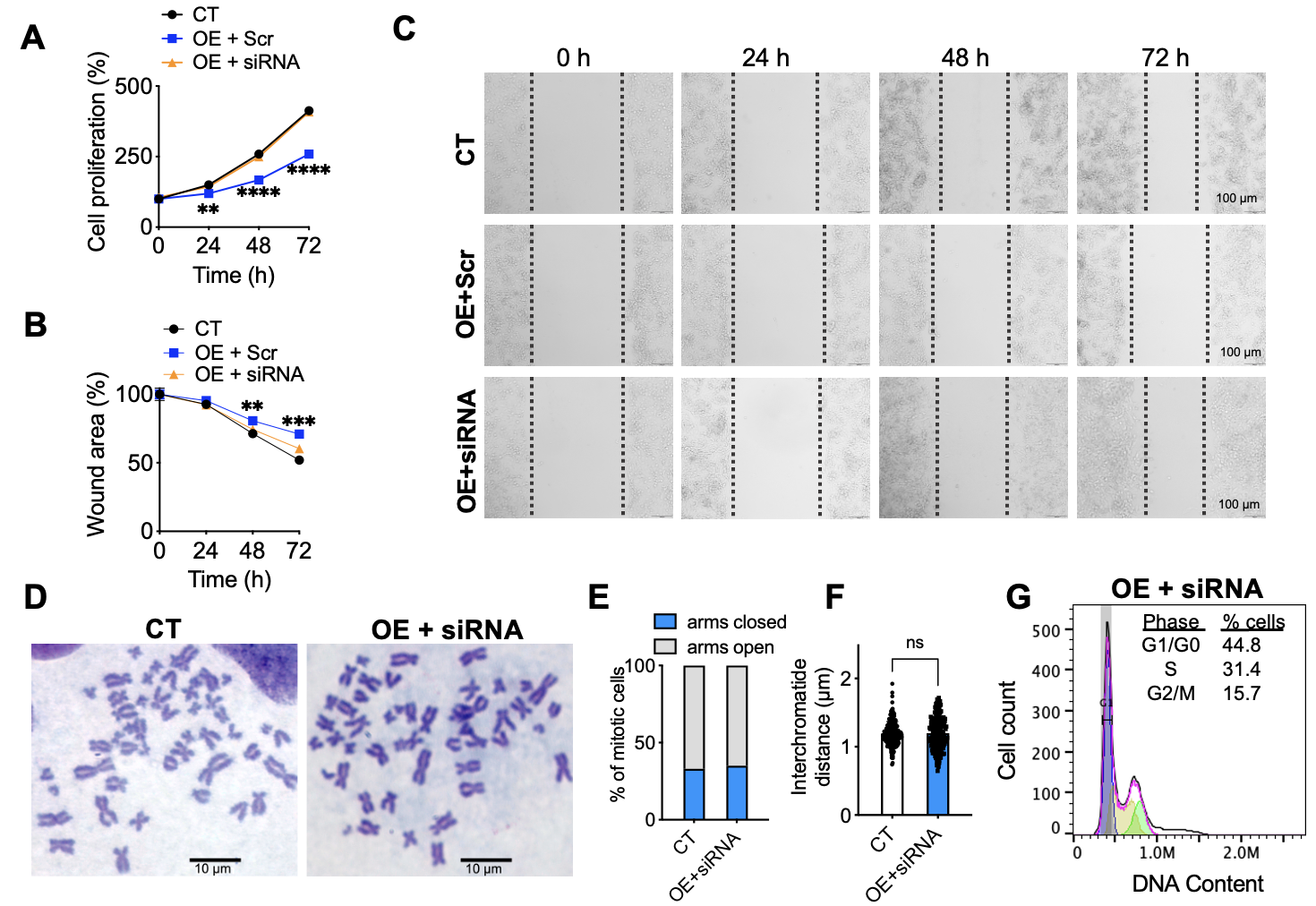

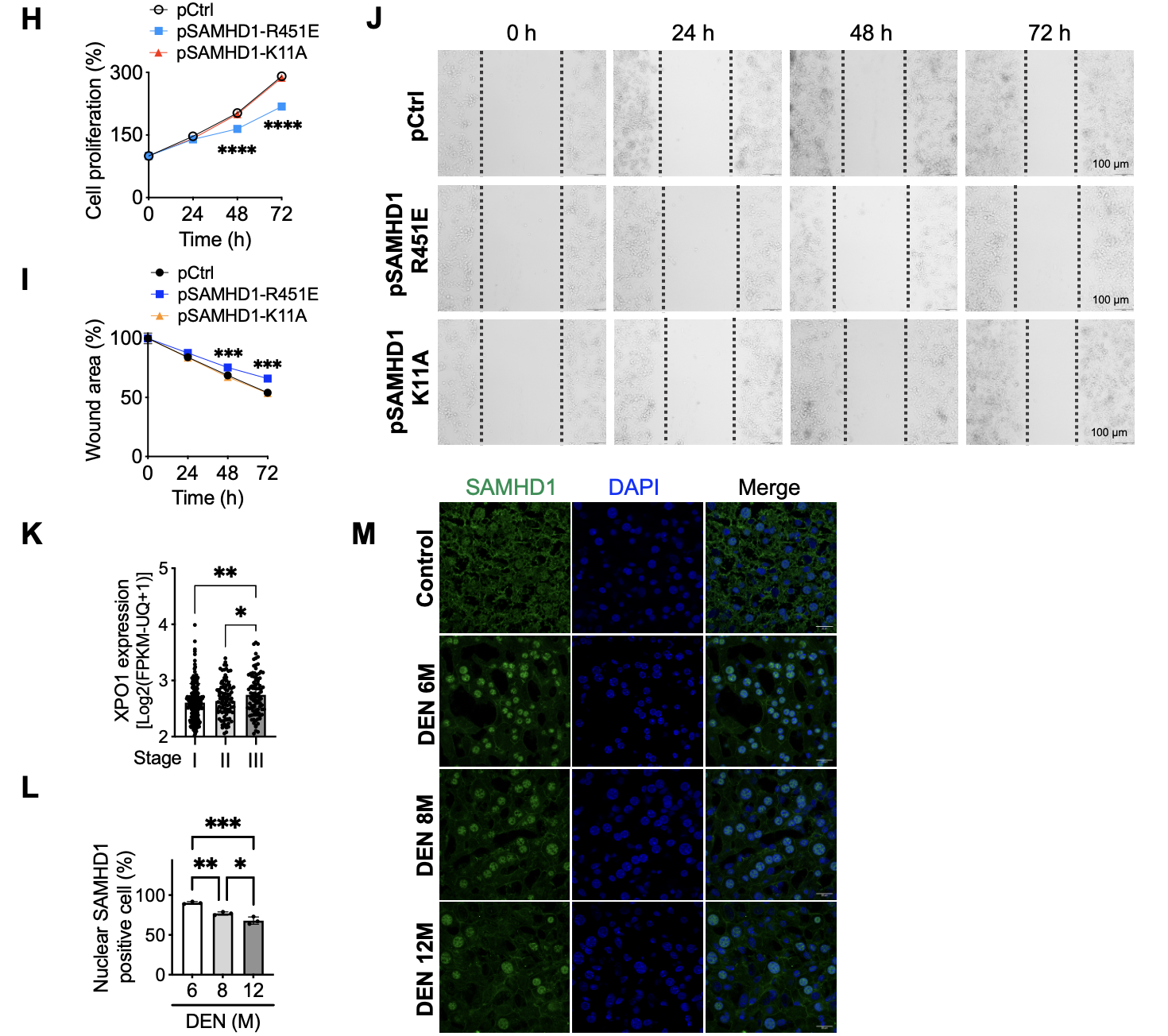


**Figure S3.** (A) CCK-8 assay results showing that the antiproliferative effect of nuclear SAMHD1 overexpression is abrogated by knockdown of cohesin complex components (n = 6 wells/group). Scr: scrambled siRNA control; siRNA: mixture of RAD21 and SMC3 siRNAs. (B, C) Representative wound healing assay images and quantification of wound closure over time in HepG2 cells, indicating that cohesin complex knockdown reduces the inhibitory effect of SAMHD1 overexpression on cell migration (n = 3 wells/group). Scale bar = 100 µm. (D) Giemsa-stained chromosome spread images of nuclear SAMHD1-overexpressing HepG2 cells with cohesin complex knockdown (OE + siRNA) compared to control cells (CT). Scale bar = 10 µm. (E) Bar graphs showing the proportion of cells with either open or closed chromosome arms. Sample sizes were n = 42 for CT and n = 55 for OE + siRNA groups. (F) Bar plot shows interchromatid distance, measured for five chromosomes across 27 randomly selected cells per group. (G) Flow cytometry analysis of PI-stained SAMHD1-overexpressing HepG2 cells after cohesin complex knockdown, showing cell cycle distribution. Cohesin complex knockdown resulted in a cell cycle distribution similar to control cells, without the increased G2/M phase accumulation observed in SAMHD1-overexpressing HepG2 cells (see Figure 6G). (H) CCK-8 assay results indicating that overexpression of the SAMHD1 dNTPase mutant R451E (pSAMHD1-R451E) inhibits HepG2 proliferation, whereas the nuclear localization sequence mutant (pSAMHD1-K11A) does not exhibit SAMHD1’s antiproliferative effect. HepG2 cells were transiently transfected with pSAMHD1-R451E, pSAMHD1-K11A, or pcDNA3.1 plasmids using Lipofectamine 3000 (n = 6 wells/group). (I, J) Overexpression of SAMHD1 R451E also reduces HepG2 cell migration, as shown in representative wound healing assay images and quantified wound closure over time (n = 3 wells/group). Scale bar = 100 µm. (K) TCGA database analysis indicates that XPO1 gene expression is significantly elevated in stage III HCC (n = 191 for Stage I, 98 for Stage II, and 97 for Stage III). (L, M) Representative immunofluorescence images of SAMHD1 staining in Flox mice at the indicated time points post-DEN induction. The percentage of nuclear SAMHD1-positive cells was quantified and presented as bar graphs (n=3/group). Flox mice at 6 months of age without DEN induction served as staining controls. Scale bar = 20 µm. Statistical analysis was conducted using Student’s t-tests in A, B, F, H, and I, and one-way ANOVA with Fisher’s LSD post hoc test in K and L to compare differences between groups. *P < 0.05, **P < 0.01, ***P < 0.001, ****P < 0.0001. Asterisks indicate the p-value for comparisons between the OE + Scr and OE + siRNA groups in A and B, and between pCtrl and pSAMHD1-R451E in H and I. Data are presented as mean ± SD.

| Target | Species | Application | Sequence (5’-3’) |
| --- | --- | --- | --- |
| SAMHD1 | Mouse | qPCR | Forward primer: CCGAGAGAAGAAAGTGCTGG  Reverse primer: ACTCCCAGATCTTCCAGACG |
| SAMHD1 | Human | qPCR | Forward primer: AGGACCACTTGAATCACCTGT  Reverse primer: GCTTTTGTTTTCAGGACGCC |
| RAD21 | Human | qPCR | Forward primer: GTGGAAAGAGACAGGAGGAGTAG  Reverse primer: AGGTCTTCTGGTACAAGCGGTG |
| SMC3 | Human | qPCR | Forward primer: ATGCGTGGAAGTCACTGCTGGA  Reverse primer: GGCAGAAAAGTAACCTCTCCAGG |
| SMC1A | Human | qPCR | Forward primer: CCTGAGACCTTCTTGCCTCTTG  Reverse primer: GAGGTGGCTCATAGCGAATCAC |
| RPS18 | Human | qPCR | Forward primer: GCAGAATCCACGCCAGTACAAG  Reverse primer: GCTTGTTGTCCAGACCATTGGC |
| β-actin | Mouse | qPCR | Forward primer: GGCTGTATTCCCCTCCATCG  Reverse primer: CCAGTTGGTAACAATGCCATGT |
| SMC3 | Human | siRNA | Sense: CAGCGGUUGGCUUUAUUGC  Anti-sense: GCAAUAAAGCCAACCGCUG |
| RAD21 | Human | siRNA | Sense: CUCCAAAUAUCUGUCAGCUAA  Anti-sense: UUAGCUGACAGAUAUUUGGAG |
| SAMHD1 | Human | sgRNA | CTCAAACACCCCTTCCGCAG |

**Table S1.** Sequence of primers, siRNA and sgRNA used in this manuscript.

| Antibody | CatLog # | Source | Application | Dilution |
| --- | --- | --- | --- | --- |
| SMC3 | ab128919 | Abcam | WB/IF | 1:1000/1:200 |
| SMC1A | ab75819 | Abcam | IF | 1:200 |
| SMC1A | sc-393171 | Santa Cruz | WB | 1:200 |
| SAMHD1 | 12586-1-AP | Proteintech | WB/IP/IHC | 1:2000/1:200/1:400 |
| SAMHD1 | ab128107 | Abcam | WB | 1/2000 |
| SAMHD1 | TA502024 | Origene | IF | 1:50 |
| RAD21 | ab217678 | Abcam | WB/IF | 1:1000/1:200 |
| Rabbit IgG | B900610 | Proteintech | IP | 1:400 |
| p21 | 10355-1-AP | Proteintech | WB | 1:2000 |
| Lamin B1 | BF8009 | Affinity Biosciences | WB | 1:1000 |
| HDAC1 | sc-81598 | Santa Cruz | WB | 1:200 |
| HA | 51064-2-AP | Proteintech | WB/IP/IF | 1:5000/1:60/1:50 |
| GAPDH | T0004 | Affinity Biosciences | WB | 1:2000 |
| E-Cadherin | 610182 | BD | WB | 1:2500 |
| Cyclin B1 | 4138 | CST | WB | 1:2000 |
| AFP | ab290637 | Abcam | IHC | 1:500 |
| HNF4⍺ | ab199431 | Abcam | IF | 1:150 |
| F4/80 | ab300421 | Abcam | IF | 1:250 |
| β-Actin | AF7018 | Affinity Biosciences | WB | 1:5000 |
| Tubulin | 66031-1-Ig | Proteintech | IF | 1:100 |
| Goat Anti-Rabbit IgG  (HRP conjugate) | E-AB-1003 | Elabscience | WB | 1:4000 |
| Goat Anti-Mouse IgG  (HRP conjugate) | E-AB-1001 | Elabscience | WB | 1:4000 |
| Goat anti-Mouse IgG (Alexa Fluor 555) | 4409 | CST | IF | 1:100 |
| Goat anti-Rabbit IgG  (Alexa Fluor 488) | ab150077 | Abcam | IF | 1:300 |

**Table S2.** Information regarding antibodies used in this manuscript.

**Supplementary information 1**

Plasmid map and nucleotide sequence of the pLVX-HA-SAMHD1-IRES-Puro plasmid, with the nuclear localization sequence coding regions highlighted in bold and underscored.


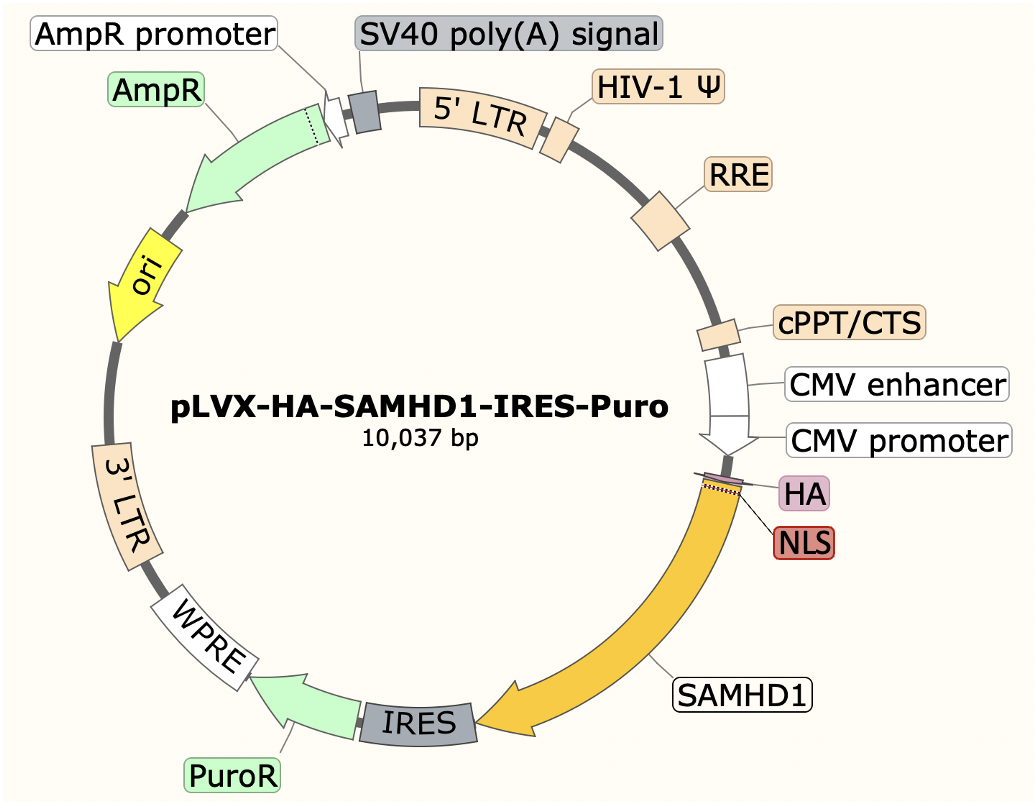


TGGAAGGGCTAATTCACTCCCAAAGAAGACAAGATATCCTTGATCTGTGGATCTACCACACACAAGGCTACTTCCCTGATTAGCAGAACTACACACCAGGGCCAGGGGTCAGATATCCACTGACCTTTGGATGGTGCTACAAGCTAGTACCAGTTGAGCCAGATAAGGTAGAAGAGGCCAATAAAGGAGAGAACACCAGCTTGTTACACCCTGTGAGCCTGCATGGGATGGATGACCCGGAGAGAGAAGTGTTAGAGTGGAGGTTTGACAGCCGCCTAGCATTTCATCACGTGGCCCGAGAGCTGCATCCGGAGTACTTCAAGAACTGCTGATATCGAGCTTGCTACAAGGGACTTTCCGCTGGGGACTTTCCAGGGAGGCGTGGCCTGGGCGGGACTGGGGAGTGGCGAGCCCTCAGATCCTGCATATAAGCAGCTGCTTTTTGCCTGTACTGGGTCTCTCTGGTTAGACCAGATCTGAGCCTGGGAGCTCTCTGGCTAACTAGGGAACCCACTGCTTAAGCCTCAATAAAGCTTGCCTTGAGTGCTTCAAGTAGTGTGTGCCCGTCTGTTGTGTGACTCTGGTAACTAGAGATCCCTCAGACCCTTTTAGTCAGTGTGGAAAATCTCTAGCAGTGGCGCCCGAACAGGGACTTGAAAGCGAAAGGGAAACCAGAGGAGCTCTCTCGACGCAGGACTCGGCTTGCTGAAGCGCGCACGGCAAGAGGCGAGGGGCGGCGACTGGTGAGTACGCCAAAAATTTTGACTAGCGGAGGCTAGAAGGAGAGAGATGGGTGCGAGAGCGTCAGTATTAAGCGGGGGAGAATTAGATCGCGATGGGAAAAAATTCGGTTAAGGCCAGGGGGAAAGAAAAAATATAAATTAAAACATATAGTATGGGCAAGCAGGGAGCTAGAACGATTCGCAGTTAATCCTGGCCTGTTAGAAACATCAGAAGGCTGTAGACAAATACTGGGACAGCTACAACCATCCCTTCAGACAGGATCAGAAGAACTTAGATCATTATATAATACAGTAGCAACCCTCTATTGTGTGCATCAAAGGATAGAGATAAAAGACACCAAGGAAGCTTTAGACAAGATAGAGGAAGAGCAAAACAAAAGTAAGACCACCGCACAGCAAGCGGCCGGCCGCTGATCTTCAGACCTGGAGGAGGAGATATGAGGGACAATTGGAGAAGTGAATTATATAAATATAAAGTAGTAAAAATTGAACCATTAGGAGTAGCACCCACCAAGGCAAAGAGAAGAGTGGTGCAGAGAGAAAAAAGAGCAGTGGGAATAGGAGCTTTGTTCCTTGGGTTCTTGGGAGCAGCAGGAAGCACTATGGGCGCAGCGTCAATGACGCTGACGGTACAGGCCAGACAATTATTGTCTGGTATAGTGCAGCAGCAGAACAATTTGCTGAGGGCTATTGAGGCGCAACAGCATCTGTTGCAACTCACAGTCTGGGGCATCAAGCAGCTCCAGGCAAGAATCCTGGCTGTGGAAAGATACCTAAAGGATCAACAGCTCCTGGGGATTTGGGGTTGCTCTGGAAAACTCATTTGCACCACTGCTGTGCCTTGGAATGCTAGTTGGAGTAATAAATCTCTGGAACAGATTTGGAATCACACGACCTGGATGGAGTGGGACAGAGAAATTAACAATTACACAAGCTTAATACACTCCTTAATTGAAGAATCGCAAAACCAGCAAGAAAAGAATGAACAAGAATTATTGGAATTAGATAAATGGGCAAGTTTGTGGAATTGGTTTAACATAACAAATTGGCTGTGGTATATAAAATTATTCATAATGATAGTAGGAGGCTTGGTAGGTTTAAGAATAGTTTTTGCTGTACTTTCTATAGTGAATAGAGTTAGGCAGGGATATTCACCATTATCGTTTCAGACCCACCTCCCAACCCCGAGGGGACCCGACAGGCCCGAAGGAATAGAAGAAGAAGGTGGAGAGAGAGACAGAGACAGATCCATTCGATTAGTGAACGGATCTCGACGGTATCGCCTTTAAAAGAAAAGGGGGGATTGGGGGGTACAGTGCAGGGGAAAGAATAGTAGACATAATAGCAACAGACATACAAACTAAAGAATTACAAAAACAAATTACAAAAATTCAAAATTTTCGGGTTTATTACAGGGACAGCAGAGATCCAGTTTATCGATAAGCTTGGGAGTTCCGCGTTACATAACTTACGGTAAATGGCCCGCCTGGCTGACCGCCCAACGACCCCCGCCCATTGACGTCAATAATGACGTATGTTCCCATAGTAACGCCAATAGGGACTTTCCATTGACGTCAATGGGTGGAGTATTTACGGTAAACTGCCCACTTGGCAGTACATCAAGTGTATCATATGCCAAGTACGCCCCCTATTGACGTCAATGACGGTAAATGGCCCGCCTGGCATTATGCCCAGTACATGACCTTATGGGACTTTCCTACTTGGCAGTACATCTACGTATTAGTCATCGCTATTACCATGGTGATGCGGTTTTGGCAGTACATCAATGGGCGTGGATAGCGGTTTGACTCACGGGGATTTCCAAGTCTCCACCCCATTGACGTCAATGGGAGTTTGTTTTGGCACCAAAATCAACGGGACTTTCCAAAATGTCGTAACAACTCCGCCCCATTGACGCAAATGGGCGGTAGGCGTGTACGGTGGGAGGTCTATATAAGCAGAGCTCGTTTAGTGAACCGTCAGATCGCCTGGAGACGCCATCCACGCTGTTTTGACCTCCATAGAAGACACCGACTCTACTAGAGGATCTATTTCCGGTGAATTCGCCACCATGTACCCATACGATGTTCCAGATTACGCTCTCGAGATGCAGCGAGCCGATTCCGAGCAGCCCTCC**AAGCGTCCCCGT**TGCGATGACAGCCCGAGAACCCCCTCAAACACCCCTTCCGCAGAGGCAGACTGGTCCCCGGGCCTGGAACTCCATCCCGACTACAAGACATGGGGTCCGGAGCAGGTGTGCTCCTTCCTCAGGCGCGGTGGCTTTGAAGAGCCGGTGCTGCTGAAGAACATCCGAGAAAATGAAATCACAGGCGCATTACTGCCTTGTCTTGATGAGTCTCGTTTTGAAAATCTTGGAGTAAGTTCCTTGGGGGAGAGGAAGAAGCTGCTTAGTTATATCCAGCGATTGGTTCAAATCCACGTTGATACAATGAAGGTAATTAATGATCCTATCCATGGCCACATTGAGCTCCACCCTCTCCTCGTCCGAATCATTGATACACCTCAATTTCAACGTCTTCGATACATCAAACAGCTGGGAGGTGGTTACTATGTTTTTCCAGGAGCTTCACACAATCGATTTGAGCATAGTCTAGGGGTGGGGTATCTAGCAGGATGTCTAGTTCACGCACTGGGTGAAAAACAACCAGAGCTGCAGATAAGTGAACGAGATGTTCTCTGTGTTCAGATTGCTGGACTTTGTCATGATCTCGGTCATGGGCCATTTTCTCACATGTTTGATGGACGATTTATTCCACTTGCTCGCCCGGAGGTGAAATGGACGCATGAACAAGGCTCAGTTATGATGTTTGAGCACCTTATTAATTCTAATGGAATTAAGCCTGTCATGGAACAATATGGTCTCATCCCTGAAGAAGATATTTGCTTTATAAAGGAACAAATTGTAGGACCACTTGAATCACCTGTCGAAGATTCATTGTGGCCATATAAAGGGCGTCCTGAAAACAAAAGCTTCCTTTATGAGATAGTATCTAATAAAAGAAATGGCATTGATGTGGACAAATGGGATTATTTTGCCAGGGACTGCCATCATCTTGGAATCCAAAATAATTTTGATTACAAGCGCTTTATTAAGTTTGCCCGTGTCTGTGAAGTAGACAATGAGTTGCGTATTTGTGCTAGAGATAAGGAAGTTGGAAATCTGTATGACATGTTCCACACTCGCAACTCTTTACACCGTAGAGCTTATCAACACAAAGTTGGCAACATTATTGATACAATGATTACAGATGCTTTCCTCAAAGCAGATGACTACATAGAGATTACAGGTGCTGGAGGAAAAAAGTATCGCATTTCTACAGCAATTGACGACATGGAAGCCTATACTAAGCTGACAGATAACATTTTTCTGGAGATTTTATACTCTACTGATCCCAAATTGAAAGACGCACGAGAGATTTTAAAACAAATTGAATACCGTAATCTATTCAAGTATGTGGGTGAGACGCAGCCAACAGGACAAATAAAGATTAAAAGGGAGGACTATGAATCTCTTCCAAAAGAGGTTGCCAGTGCTAAACCCAAAGTATTGCTAGACGTGAAACTGAAGGCTGAAGATTTTATAGTGGATGTTATCAACATGGATTATGGAATGCAAGAAAAGAATCCAATTGATCATGTTAGCTTCTATTGTAAGACTGCCCCCAACAGAGCAATCAGGATTACTAAAAACCAGGTTTCACAACTTCTGCCAGAGAAATTTGCAGAGCAGCTGATTCGAGTATATTGTAAGAAGGTGGACAGAAAGAGTTTGTATGCCGCAAGACAATATTTTGTTCAGTGGTGTGCAGACAGAAATTTCACCAAGCCGCAGGATGGCGATGTTATAGCCCCACTCATAACACCTCAAAAAAAGGAATGGAACGACAGTACTTCAGTCCAAAATCCAACTCGCCTCCGAGAAGCATCCAAAAGCAGAGTCCAGCTTTTTAAAGATGACCCAATGTGAGGATCCCGCCCCTCTCCCTCCCCCCCCCCTAACGTTACTGGCCGAAGCCGCTTGGAATAAGGCCGGTGTGCGTTTGTCTATATGTTATTTTCCACCATATTGCCGTCTTTTGGCAATGTGAGGGCCCGGAAACCTGGCCCTGTCTTCTTGACGAGCATTCCTAGGGGTCTTTCCCCTCTCGCCAAAGGAATGCAAGGTCTGTTGAATGTCGTGAAGGAAGCAGTTCCTCTGGAAGCTTCTTGAAGACAAACAACGTCTGTAGCGACCCTTTGCAGGCAGCGGAACCCCCCACCTGGCGACAGGTGCCTCTGCGGCCAAAAGCCACGTGTATAAGATACACCTGCAAAGGCGGCACAACCCCAGTGCCACGTTGTGAGTTGGATAGTTGTGGAAAGAGTCAAATGGCTCTCCTCAAGCGTATTCAACAAGGGGCTGAAGGATGCCCAGAAGGTACCCCATTGTATGGGATCTGATCTGGGGCCTCGGTGCACATGCTTTACATGTGTTTAGTCGAGGTTAAAAAAACGTCTAGGCCCCCCGAACCACGGGGACGTGGTTTTCCTTTGAAAAACACGATGATAAGCTTGCCACAACCCACAAGGAGACGACCTTCCATGACCGAGTACAAGCCCACGGTGCGCCTCGCCACCCGCGACGACGTCCCCCGGGCCGTACGCACCCTCGCCGCCGCGTTCGCCGACTACCCCGCCACGCGCCACACCGTCGACCCGGACCGCCACATCGAGCGGGTCACCGAGCTGCAAGAACTCTTCCTCACGCGCGTCGGGCTCGACATCGGCAAGGTGTGGGTCGCGGACGACGGCGCCGCGGTGGCGGTCTGGACCACGCCGGAGAGCGTCGAAGCGGGGGCGGTGTTCGCCGAGATCGGCCCGCGCATGGCCGAGTTGAGCGGTTCCCGGCTGGCCGCGCAGCAACAGATGGAAGGCCTCCTGGCGCCGCACCGGCCCAAGGAGCCCGCGTGGTTCCTGGCCACCGTCGGCGTCTCGCCCGACCACCAGGGCAAGGGTCTGGGCAGCGCCGTCGTGCTCCCCGGAGTGGAGGCGGCCGAGCGCGCCGGGGTGCCCGCCTTCCTGGAGACCTCCGCGCCCCGCAACCTCCCCTTCTACGAGCGGCTCGGCTTCACCGTCACCGCCGACGTCGAGGTGCCCGAAGGACCGCGCACCTGGTGCATGACCCGCAAGCCCGGTGCCTAGACGCGTCTGGAACAATCAACCTCTGGATTACAAAATTTGTGAAAGATTGACTGGTATTCTTAACTATGTTGCTCCTTTTACGCTATGTGGATACGCTGCTTTAATGCCTTTGTATCATGCTATTGCTTCCCGTATGGCTTTCATTTTCTCCTCCTTGTATAAATCCTGGTTGCTGTCTCTTTATGAGGAGTTGTGGCCCGTTGTCAGGCAACGTGGCGTGGTGTGCACTGTGTTTGCTGACGCAACCCCCACTGGTTGGGGCATTGCCACCACCTGTCAGCTCCTTTCCGGGACTTTCGCTTTCCCCCTCCCTATTGCCACGGCGGAACTCATCGCCGCCTGCCTTGCCCGCTGCTGGACAGGGGCTCGGCTGTTGGGCACTGACAATTCCGTGGTGTTGTCGGGGAAGCTGACGTCCTTTCCATGGCTGCTCGCCTGTGTTGCCACCTGGATTCTGCGCGGGACGTCCTTCTGCTACGTCCCTTCGGCCCTCAATCCAGCGGACCTTCCTTCCCGCGGCCTGCTGCCGGCTCTGCGGCCTCTTCCGCGTCTTCGCCTTCGCCCTCAGACGAGTCGGATCTCCCTTTGGGCCGCCTCCCCGCCTGGAATTAATTCTGCAGTCGAGACCTAGAAAAACATGGAGCAATCACAAGTAGCAATACAGCAGCTACCAATGCTGATTGTGCCTGGCTAGAAGCACAAGAGGAGGAGGAGGTGGGTTTTCCAGTCACACCTCAGGTACCTTTAAGACCAATGACTTACAAGGCAGCTGTAGATCTTAGCCACTTTTTAAAAGAAAAGAGGGGACTGGAAGGGCTAATTCACTCCCAACGAAGACAAGATATCCTTGATCTGTGGATCTACCACACACAAGGCTACTTCCCTGATTAGCAGAACTACACACCAGGGCCAGGGGTCAGATATCCACTGACCTTTGGATGGTGCTACAAGCTAGTACCAGTTGAGCCAGATAAGGTAGAAGAGGCCAATAAAGGAGAGAACACCAGCTTGTTACACCCTGTGAGCCTGCATGGGATGGATGACCCGGAGAGAGAAGTGTTAGAGTGGAGGTTTGACAGCCGCCTAGCATTTCATCACGTGGCCCGAGAGCTGCATCCGGAGTACTTCAAGAACTGCTGATATCGAGCTTGCTACAAGGGACTTTCCGCTGGGGACTTTCCAGGGAGGCGTGGCCTGGGCGGGACTGGGGAGTGGCGAGCCCTCAGATCCTGCATATAAGCAGCTGCTTTTTGCCTGTACTGGGTCTCTCTGGTTAGACCAGATCTGAGCCTGGGAGCTCTCTGGCTAACTAGGGAACCCACTGCTTAAGCCTCAATAAAGCTTGCCTTGAGTGCTTCAAGTAGTGTGTGCCCGTCTGTTGTGTGACTCTGGTAACTAGAGATCCCTCAGACCCTTTTAGTCAGTGTGGAAAATCTCTAGCAGTAGTAGTTCATGTCATCTTATTATTCAGTATTTATAACTTGCAAAGAAATGAATATCAGAGAGTGAGAGGCCTTGACATTGCTAGCGTTTACCGTCGACCTCTAGCTAGAGCTTGGCGTAATCATGGTCATAGCTGTTTCCTGTGTGAAATTGTTATCCGCTCACAATTCCACACAACATACGAGCCGGAAGCATAAAGTGTAAAGCCTGGGGTGCCTAATGAGTGAGCTAACTCACATTAATTGCGTTGCGCTCACTGCCCGCTTTCCAGTCGGGAAACCTGTCGTGCCAGCTGCATTAATGAATCGGCCAACGCGCGGGGAGAGGCGGTTTGCGTATTGGGCGCTCTTCCGCTTCCTCGCTCACTGACTCGCTGCGCTCGGTCGTTCGGCTGCGGCGAGCGGTATCAGCTCACTCAAAGGCGGTAATACGGTTATCCACAGAATCAGGGGATAACGCAGGAAAGAACATGTGAGCAAAAGGCCAGCAAAAGGCCAGGAACCGTAAAAAGGCCGCGTTGCTGGCGTTTTTCCATAGGCTCCGCCCCCCTGACGAGCATCACAAAAATCGACGCTCAAGTCAGAGGTGGCGAAACCCGACAGGACTATAAAGATACCAGGCGTTTCCCCCTGGAAGCTCCCTCGTGCGCTCTCCTGTTCCGACCCTGCCGCTTACCGGATACCTGTCCGCCTTTCTCCCTTCGGGAAGCGTGGCGCTTTCTCATAGCTCACGCTGTAGGTATCTCAGTTCGGTGTAGGTCGTTCGCTCCAAGCTGGGCTGTGTGCACGAACCCCCCGTTCAGCCCGACCGCTGCGCCTTATCCGGTAACTATCGTCTTGAGTCCAACCCGGTAAGACACGACTTATCGCCACTGGCAGCAGCCACTGGTAACAGGATTAGCAGAGCGAGGTATGTAGGCGGTGCTACAGAGTTCTTGAAGTGGTGGCCTAACTACGGCTACACTAGAAGAACAGTATTTGGTATCTGCGCTCTGCTGAAGCCAGTTACCTTCGGAAAAAGAGTTGGTAGCTCTTGATCCGGCAAACAAACCACCGCTGGTAGCGGTGGTTTTTTTGTTTGCAAGCAGCAGATTACGCGCAGAAAAAAAGGATCTCAAGAAGATCCTTTGATCTTTTCTACGGGGTCTGACGCTCAGTGGAACGAAAACTCACGTTAAGGGATTTTGGTCATGAGATTATCAAAAAGGATCTTCACCTAGATCCTTTTAAATTAAAAATGAAGTTTTAAATCAATCTAAAGTATATATGAGTAAACTTGGTCTGACAGTTACCAATGCTTAATCAGTGAGGCACCTATCTCAGCGATCTGTCTATTTCGTTCATCCATAGTTGCCTGACTCCCCGTCGTGTAGATAACTACGATACGGGAGGGCTTACCATCTGGCCCCAGTGCTGCAATGATACCGCGAGACCCACGCTCACCGGCTCCAGATTTATCAGCAATAAACCAGCCAGCCGGAAGGGCCGAGCGCAGAAGTGGTCCTGCAACTTTATCCGCCTCCATCCAGTCTATTAATTGTTGCCGGGAAGCTAGAGTAAGTAGTTCGCCAGTTAATAGTTTGCGCAACGTTGTTGCCATTGCTACAGGCATCGTGGTGTCACGCTCGTCGTTTGGTATGGCTTCATTCAGCTCCGGTTCCCAACGATCAAGGCGAGTTACATGATCCCCCATGTTGTGCAAAAAAGCGGTTAGCTCCTTCGGTCCTCCGATCGTTGTCAGAAGTAAGTTGGCCGCAGTGTTATCACTCATGGTTATGGCAGCACTGCATAATTCTCTTACTGTCATGCCATCCGTAAGATGCTTTTCTGTGACTGGTGAGTACTCAACCAAGTCATTCTGAGAATAGTGTATGCGGCGACCGAGTTGCTCTTGCCCGGCGTCAATACGGGATAATACCGCGCCACATAGCAGAACTTTAAAAGTGCTCATCATTGGAAAACGTTCTTCGGGGCGAAAACTCTCAAGGATCTTACCGCTGTTGAGATCCAGTTCGATGTAACCCACTCGTGCACCCAACTGATCTTCAGCATCTTTTACTTTCACCAGCGTTTCTGGGTGAGCAAAAACAGGAAGGCAAAATGCCGCAAAAAAGGGAATAAGGGCGACACGGAAATGTTGAATACTCATACTCTTCCTTTTTCAATATTATTGAAGCATTTATCAGGGTTATTGTCTCATGAGCGGATACATATTTGAATGTATTTAGAAAAATAAACAAATAGGGGTTCCGCGCACATTTCCCCGAAAAGTGCCACCTGACGTCGACGGATCGGGAGATCAACTTGTTTATTGCAGCTTATAATGGTTACAAATAAAGCAATAGCATCACAAATTTCACAAATAAAGCATTTTTTTCACTGCATTCTAGTTGTGGTTTGTCCAAACTCATCAATGTATCTTATCATGTCTGGATCAACTGGATAACTCAAGCTAACCAAAATCATCCCAAACTTCCCACCCCATACCCTATTACCACTGCCAATTACCTGTGGTTTCATTTACTCTAAACCTGTGATTCCTCTGAATTATTTTCATTTTAAAGAAATTGTATTTGTTAAATATGTACTACAAACTTAGTAGTTTTTAAAGAAATTGTATTTGTTAAATATGTACTACAAACTTAGTAG
